# High-density lipoprotein cholesterol as a prognostic marker for 90-day transplant-free mortality in hepatitis B virus-related acute-on-chronic liver failure

**DOI:** 10.3389/fcimb.2024.1458818

**Published:** 2025-01-22

**Authors:** Ke Shi, Yi Zhang, Yanqiu Li, Xiaojing Wang, Ying Feng, Xianbo Wang

**Affiliations:** Center of Integrative Medicine, Beijing Ditan Hospital, Capital Medical University, Beijing, China

**Keywords:** hepatitis B virus, high-density lipoprotein cholesterol, acute-on-chronic liver failure, inflammatory factor, mortality, prognosis

## Abstract

**Background:**

Hepatitis B virus-related acute-on-chronic liver failure (HBV-ACLF) is linked to dyslipidemia and inflammatory responses. This study aimed to investigate the correlation between high-density lipoprotein cholesterol (HDL-C) levels and 90-day transplant-free (TF) mortality in patients with HBV-ACLF.

**Methods:**

A prospective cohort of 287 patients with HBV-ACLF from Beijing Ditan Hospital was enrolled between January 2016 and December 2019. The prognostic accuracy of lipid profile parameters was evaluated by the area under the receiver operating characteristic curve (AUC), and the association between HDL-C levels and mortality was assessed using a restricted cubic spline analysis. Correlations between lipid profile parameters and inflammatory factors were analyzed. Kaplan–Meier curves were used to assess 90-day TF mortality, and log-rank tests were used for comparison analysis. These results were internally validated between January 2020 and December 2023 (n=125).

**Results:**

Patients with lower HDL-C levels exhibited higher mortality rates (adjusted hazard ratio for HDL-C < 0.13 mmol/L: 4.04, 95% confidence interval: 1.35–11.85) compared with those in the reference group (with HDL-C levels above 0.36 mmol/L). An “L-shaped” association was observed between HDL-C levels and TF mortality. The prognostic value of HDL-C (AUC at day 90: 0.732) was comparable to the model for end-stage liver disease score of 0.729. Additionally, HDL-C levels were inversely correlated with interleukin (IL)-4, IL-6, and tumor necrosis factor-α (all *P*<0.05). In the training cohort, the 90-day TF mortality rates were 8.3%, 15.2%, 24.0%, and 43.2% for the extremely low, low, medium, and high-risk subgroups, respectively, while in the validation cohort, they were 4.5%, 18.5%, 31.2%, and 44.7%, respectively.

**Conclusions:**

HDL-C levels < 0.13 mmol/L were associated with increased 90-day transplant-free mortality in patients with HBV-ACLF. An inverse correlation was found between HDL-C levels and inflammatory markers.

## Introduction

Acute-on-chronic liver failure (ACLF) imposes substantial morbidity and mortality, with mortality rates ranging from 20–30% at 28 days to 50–70% at 6 months (Moreau et al., 2021; Bajaj et al., 2022). In the Asia-Pacific region, hepatitis B virus (HBV)-related ACLF (HBV-ACLF) is the most common form (Li et al., 2021). Systemic inflammation and immune disorders play critical roles in the progression of HBV-ACLF (Wong et al., 2021). Currently, there are no specific drugs for the treatment of ACLF. Therefore, accurate early prediction of short-term prognosis in HBV-ACLF is crucial for making timely and appropriate liver transplantation decisions.

Dysfunctional high-density lipoprotein (HDL) has been implicated in inflammatory disorders such as cardiovascular diseases, infections, malignancies, diabetes, and kidney disease (Wargny et al., 2024; Casula et al., 2021; Pei et al., 2022; Young et al., 2019). Given the liver’s pivotal role in lipid production, particularly in lipoprotein synthesis (Zhang et al., 2023), recent studies have noted the frequent development of hypocholesterolemia in patients with cirrhosis (He et al., 2021). HDL cholesterol (HDL-C) levels were markedly decreased in patients with liver failure, and the extent of the decrease was associated with poor prognosis, suggesting that HDL-C may participate in the immune and inflammatory response of the body, offering potential diagnostic and predictive value (Trieb et al., 2020). Research indicates that incorporating HDL-C into the Model for End-Stage Liver Disease (MELD) score enhances short-term prognostic accuracy in patients with cirrhosis (Wang et al., 2020). Additionally, serum lipid levels and significant inflammatory responses are implicated in the pathogenesis of ACLF (Bajaj et al., 2020; Clàri et al., 2016). During chronic liver disease or infection, HDL loses its protective functions, leading to HDL dysfunction, such as reduced cholesterol efflux capacity, regulation of endothelial cell apoptosis, and reduced nitric oxide production (Thabut et al., 2007). However, the relationship between lipid levels and inflammatory markers in patients with HBV-ACLF, as well as a method to identify high-risk patients based on different HDL-C levels, remain unclear.

This study aimed to assess the predictive value of HDL-C levels for 90-day TF mortality and identify high-risk patients, while concurrently exploring the relationship between HDL-C and inflammatory markers in patients with HBV-ACLF.

## Materials and methods

### Participants

**Figure 1:**
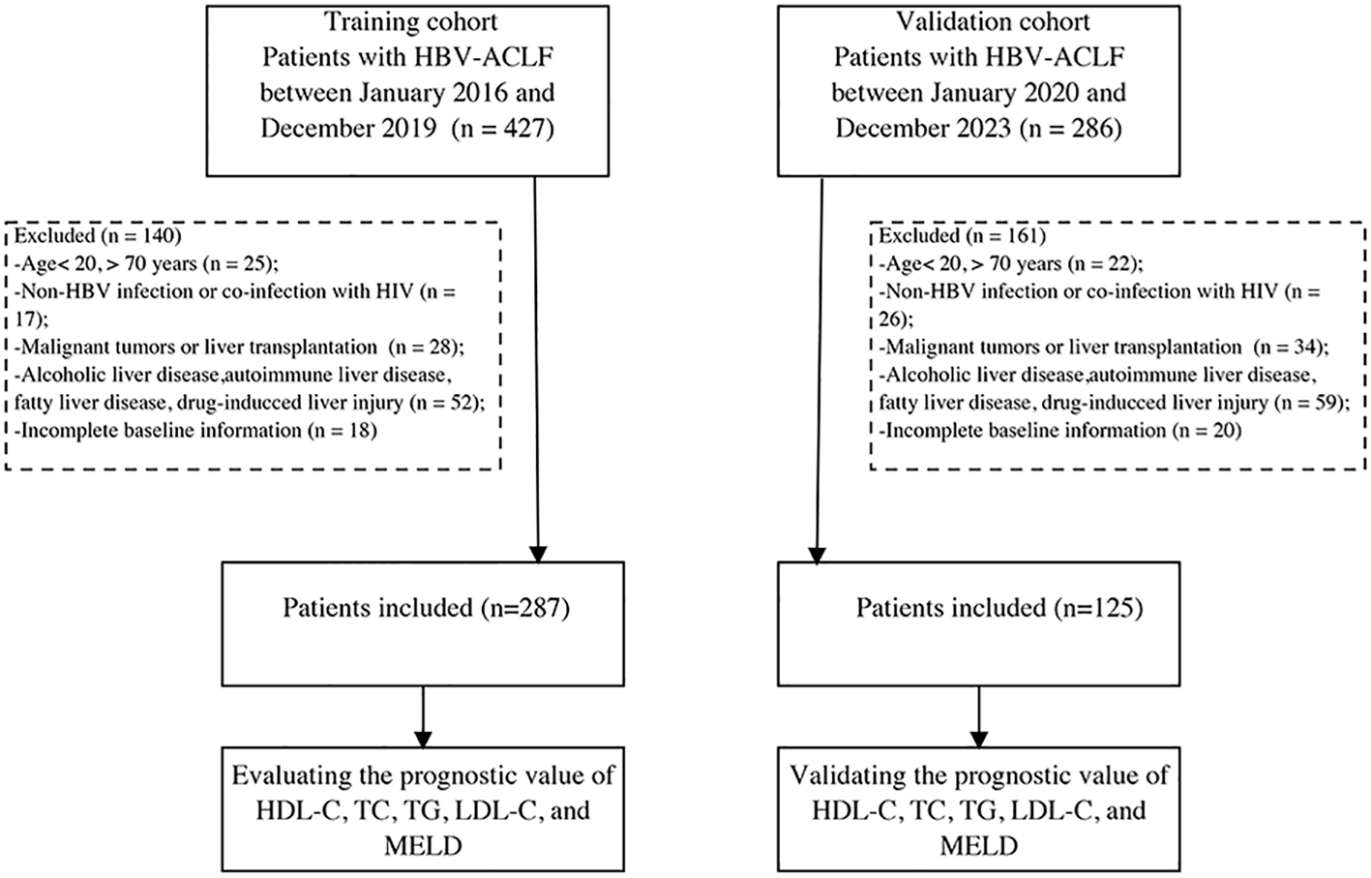
Study flow diagram.

Between January 2016 and December 2019, we prospectively screened 287 patients with HBV-ACLF admitted to Beijing Ditan Hospital as a training cohort, following the diagnostic guidelines of the Asia Pacific Association for the Study of the Liver for ACLF (Sarin et al., 2019). Chronic hepatitis B was defined as the presence of hepatitis B surface antigenemia for over 6 months (Sarin et al., 2016). The diagnosis of cirrhosis was based on histological, endoscopic, or imaging findings (ultrasound, computed tomography, or magnetic resonance imaging), and signs of portal hypertension (Shiha et al., 2009). Exclusion criteria included: i) age below 20 or above 70 years; ii) viral hepatitis due to other causes than HBV or co-infection with human immunodeficiency virus; iii) presence of malignant tumors or history of impending liver transplantation; iv) fatty liver disease, drug-induced liver injury, alcoholic liver disease, cholestatic disorders, inherited liver conditions, or autoimmune liver disease; and v) incomplete baseline information. Additionally, 125 patients were prospectively included as a validation cohort from January 2020 to December 2023 using the same criteria (**Figure 1**). To compare inflammatory factors with those in patients with HBV-ACLF, we included a control group of 20 healthy individuals without liver or other diseases. The study protocol conformed to the ethical guidelines of the 1975 Declaration of Helsinki and was approved by the Ethics Committee of Beijing Ditan Hospital. Written informed consent was obtained from all participants.

### Data collection and measurement of plasma cytokines

Baseline data, including demographics, complications, and laboratory tests, were collected, and mortality information at 90 days of follow-up was recorded via telephone or outpatient visits. The MELD score was calculated according to the published formula (Pommergaard et al., 2021). Plasma from 67 HBV-ACLF patients from January to December 2019 and 33 HBV-ACLF patients from April to October 2023 were selected for analysis. Plasma samples were obtained from 100 patients with HBV-ACLF (training cohort, n = 67; validation cohort, n = 33) and 20 healthy controls, and centrifuged at 1,000 ×g for 10 minutes at 4°C. Levels of tumor necrosis factor-α (TNF-α), interferon-γ (IFN-γ), interleukin (IL)-1β, IL-8, IL-6, and IL-4 were measured using enzyme-linked immunosorbent assay kits according to the manufacturer’s protocols (R&D Systems, Minneapolis, MN, USA).

### Statistical analysis

Quantitative data are expressed as mean ± standard deviation or median (interquartile range) and categorical data as frequencies or percentages. Continuous variables were compared using the Mann-Whitney U test or Student’s *t*-test, and categorical variables using the chi-square test or Fisher’s exact test. Multivariate Cox regression analysis was performed to identify risk factors, with hazard ratios (HRs) and 95% confidence intervals (CIs) estimated for outcome events. A *P* value < 0.05 was considered statistically significant. Data analyses were conducted using SPSS version 25.0 (SPSS, Inc., Chicago, IL, USA).

Restricted cubic spline (RCS) analysis with 3 knots at the 10th, 50th, and 90th percentiles of HDL-C levels was employed to flexibly model the association between HDL-C and prognosis. To assess the importance of inclusion indicators, random forest analysis with tenfold cross-verification was utilized. The prognostic accuracy of lipid profile indicators and the MELD score was evaluated using area under receiver operating characteristic curve (AUC). The AUC comparisons were conducted using the Delong test. A forest plot was used to illustrate the relationship between HDL-C and prognosis in different subgroups. The correlations between lipid profile parameters and inflammatory markers were explored using Spearman’s correlation analysis. The Kaplan-Meier method was used to estimate differences in 90-day TF mortality rates, which were compared using the log-rank test. The statistical analyses were performed using R (version 4.2.3; R Foundation, Nashville, TN, USA).

## Results

### Patient characteristics

**Figure 2:**
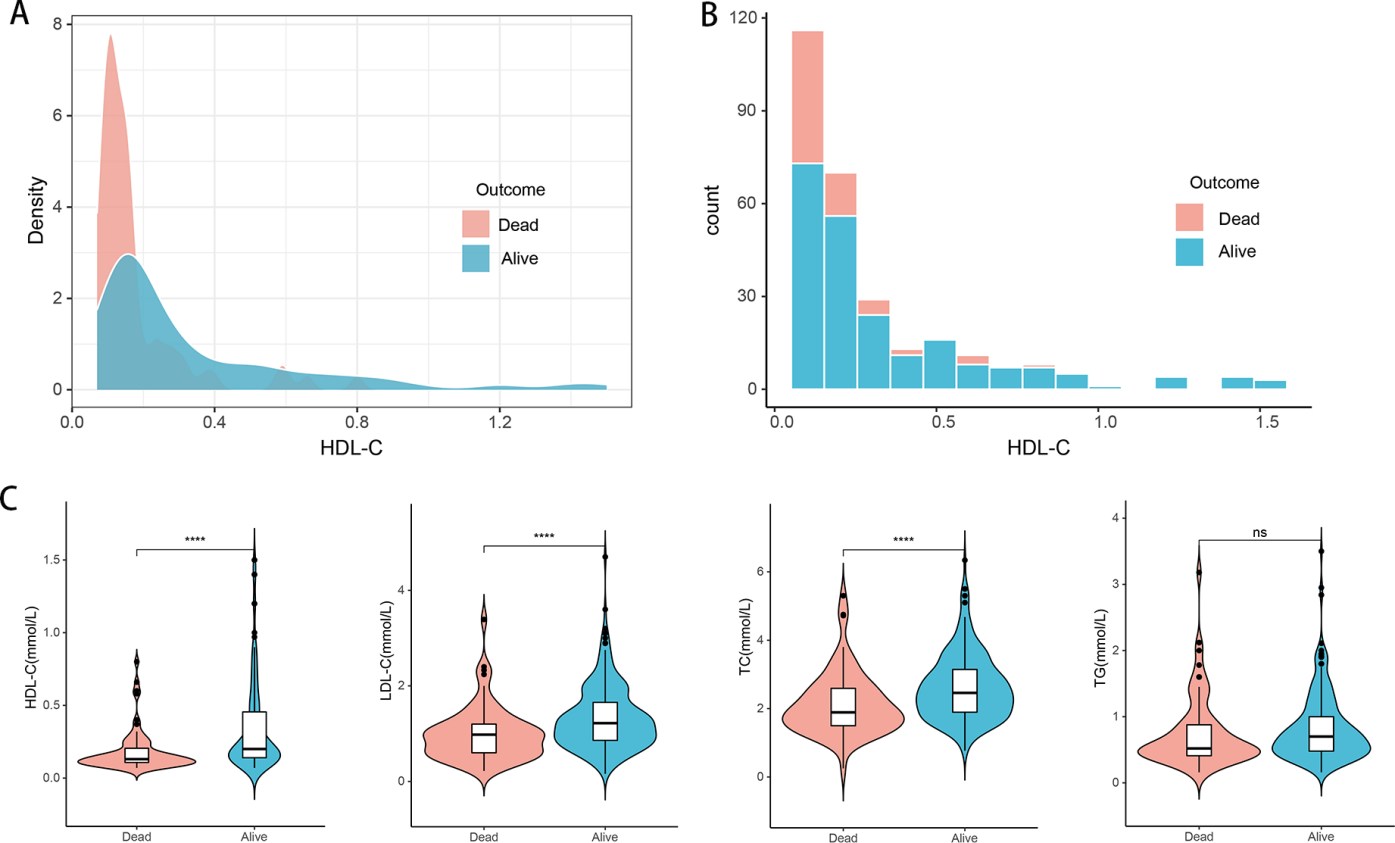
Distribution of lipid-related indicators in all HBV-ACLF patients (n=412) with survival (n=315) and death (n=97). **(A, B)** Density map and histogram of high-density lipoprotein cholesterol in survival (n=315) and death (n=97) patients. **(C)** Differences of HDL-C, LDL-C, TC, and TG between (n=315) and death (n=97) patients. TC, total cholesterol; TG, triglyceride; HDL-C, high-density lipoprotein cholesterol; LDL-C, low-density lipoprotein cholesterol; HBV-ACLF, hepatitis B virus related acute-on-chronic liver failure. ****P<0.0001; ns: not significant (P > 0.05).

The median age of the patients was 46 years (range 40–55 years). Of these patients, 231 (80.5%) were male, and 245 (85.3%) were diagnosed with cirrhosis. Compared with survivors, non-survivors were older, had a higher incidence of hepatic encephalopathy (HE), elevated total bilirubin (TBIL) levels, higher neutrophil-to-lymphocyte ratio (NLR), higher MELD scores, and higher international normalized ratio (INR) (**Table 1**). In contrast, non-survivors exhibited lower levels of total cholesterol (TC), low-density lipoprotein cholesterol (LDL-C), and HDL-C (**Figures 2A–C**). During the follow-up period, there were 68 (23.7%) deaths in the training cohort and 29 (23.2%) deaths in the validation cohort. There were no significant differences in clinical and laboratory parameters between the two groups.

**Table 1:** Clinical baseline characteristics of patients with HBV-ACLF in the training and the validation cohorts.

Variables	Training cohort (n = 287)	Survived (n = 219)	Death (n = 68)	Validation cohort (n = 125)	Survived (n = 96)	Death (n = 29)	*P*-value*
Age (years)	46.0 (40.0, 55.0)	46.0 (37.0, 54.0)	49.0 (42.0, 57.0)	46.0 (36.0, 53.0)	46.0 (36.0, 52.0)	48.0 (39.0, 57.0)	0.075
Male (%)	231 (80.5)	178 (81.3)	53 (77.9)	102 (81.6)	77 (80.2)	25 (86.2)	0.792
Cirrhosis (%)	245 (85.3)	181 (82.6)	64 (94.1)	101 (80.8)	77 (80.2)	24 (82.7)	0.335
Ascites (%)	193 (67.2)	143 (65.3)	50 (73.5)	78 (62.4)	57 (59.3)	21 (72.4)	0.340
GIB (%)							
HE (%)	73 (25.4)	44 (20.1)	29 (42.6)	27 (21.6)	15 (15.6)	12 (41.3)	0.404
ALT (IU/L)	117.5 (46.3, 522.8)	128.0 (48.1, 563.0)	102.6(39.3, 503.2)	182.3(163.1, 663.6)	171.4 (59.0, 617.1)	327.1(69.3, 773.2)	0.882
AST (IU/L)	132.5 (60.3, 338.2)	131.7 (60.2, 349.2)	142.4(60.8, 288.0)	197.4 (76.1, 457.7)	185.8 (75.6, 479.8)	218.5(95.6, 489.8)	0.229
TBIL (µmol/L)	206.1 (118.7, 294.9)	185.5 (113.1, 261.2)	258.4 (144.4, 384.5)	198.6 (117.1, 323.6)	178.7 (106.9, 296.1)	321.4 (222.0, 387.4)	0.249
Albumin (g/L)	32.0 ± 8.6	32.5 ± 9.0	30.6 ± 7.1	33.9 ± 9.6	34.2 ± 9.7	30.4 ± 9.4	0.062
TC (mmol/L)	2.4 (1.7, 3.0)	2.5 (1.9, 3.2)	1.8 (1.5, 2.6)	2.4 (1.9, 3.2)	2.5 (1.9, 3.5)	2.0 (1.3, 2.6)	0.263
TG (mmol/L)	0.6 (0.5, 1.0)	0.7 (0.5, 1.0)	0.5 (0.4, 0.9)	0.7 (0.5, 1.2)	0.7 (0.4, 1.2)	0.7 (0.4, 1.2)	0.103
HDL-C(mmol/L)	0.2 (0.1,0.3)	0.2 (0.1, 0.5)	0.1 (0.1, 0.2)	0.2 (0.1, 0.4)	0.3 (0.1, 0.6)	0.1 (0.1, 0.2)	0.103
LDL-C (mmol/L)	1.1 (0.8, 1.6)	1.2 (0.8, 1.7)	1.0 (0.5, 1.2)	1.1 (0.7, 1.6)	1.1 (0.7, 1.6)	1.1 (0.7, 1.6)	0.782
WBC (×10^9^/L)	5.7 (3.7, 7.6)	5.4 (3.9, 7.4)	6.3 (4.1, 8.0)	5.2 (4.1, 7.2)	5.1 (4.2, 7.2)	5.2 (3.7, 7.3)	0.408
NLR	2.8 (1.7, 4.9)	2.7 (1.8, 4.5)	3.5 (2.4, 6.0)	2.7 (1.8, 3.9)	2.5 (1.6, 3.6)	3.5 (2.5, 5.5)	0.528
PLT (×10^9^/L)	89.0 (57.0, 133.0)	89.0 (59.0, 133.0)	87.7 (57.1, 133.5)	93.0 (64.2, 123.2)	93.7 (62.7, 124.9)	92.0 (65.3, 120.3)	0.901
INR	2.0 (1.8, 2.4)	2.0 (1.8, 2.4)	2.1 (1.8, 2.7)	1.9 (1.7, 2.4)	1.9 (1.6, 2.2)	2.3 (1.7, 3.0)	0.905
Cr (µmol/L)	66.0 (57.0, 79.0)	66.2 (57.0, 77.8)	66.3 (55.8, 84.6)	65.3 (56.6, 79.2)	65.0 (57.2, 76.3)	70.4 (55.5, 86.0)	0.743
Na (mmol/L)	137.0 (133.5, 139.9)	137.6(134.6, 140.1)	134.6(130.4, 137.3)	138.0 (135.4, 140.0)	138.6 (136.0, 140.1)	137.0 (133.8, 139.2)	0.101
MELD score	24.4 (20.8, 27.4)	23.4 (20.7, 26.7)	27.3 (24.2, 30.9)	23.2 (20.9, 26.8)	22.9 (20.3, 25.5)	27.4 (22.8, 32.1)	0.455
Mortality (%)	68 (23.7)	–	–	29 (23.2)	–	–	0.914

Data are presented as n (%), mean ± SD, or median (interquartile range). ALT, alanine aminotransferase; AST, aspartate aminotransferase; TBIL, total bilirubin; ALB,albumin; PLT, platelet count; TC, total cholesterol; TG,triglyceride; HDL-C, high-density lipoprotein cholesterol; LDL-C, low-density lipoprotein cholesterol; HE, hepatic encephalopathy; HBV, hepatitis B virus; WBC, white blood cell; NLR, neutrophil-lymphocyte ratio; GIB, gastrointestinal bleeding; INR, international normalized ratio; Cr, creatinine; MELD, model for end-stage liver disease; HBV-ACLF, hepatitis B virus related acute-on-chronic liver failure.

*The *P*-value represents the comparison between the training set and the test set.

### Prognostic value of HDL-C in patients with HBV-ACLF

**Figure 3:**
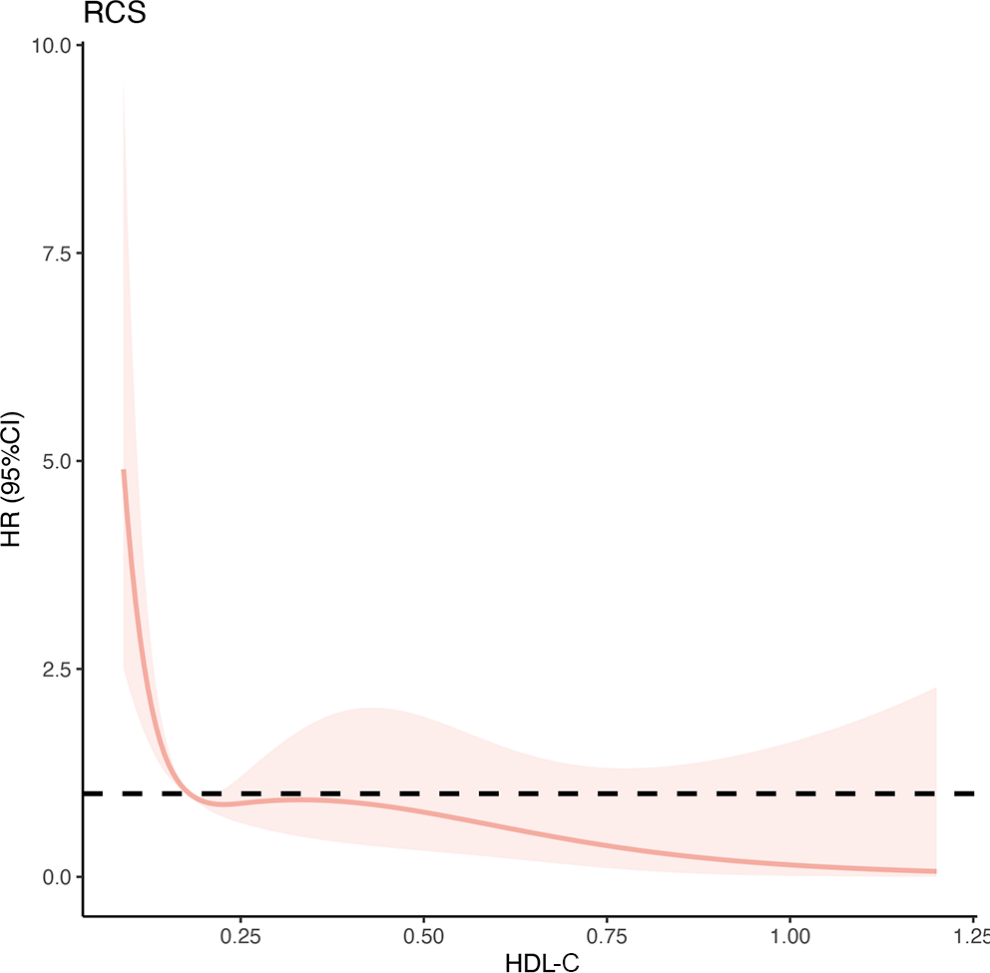
Multivariable-adjusted hazard ratios of 90-day transplant-free mortality in patients with HBV-ACLF in the training cohort (n=287). The red solid lines indicate multivariate-adjusted hazard ratios and the dash area indicate the 95% CIs. The Cox regression was adjusted for age, sex, ascites, gastrointestinal bleeding, hepatic encephalopathy, total bilirubin, total cholesterol, neutrophil-lymphocyte ratio, international normalized ratio, and creatinine. HBV-ACLF, hepatitis B virus related acute-on-chronic liver failure.

A random forest analysis was performed to rank the importance of clinical variables, and HDL-C was second only to MELD score after 10-fold cross-validation (**Figures 3A, B**). The prognostic values of HDL-C at day 28 and day 90 were evaluated using receiver operating curves (**Figures 3C–F**). In the training cohort, the AUC of HDL-C for 28-day TF mortality was 0.744 (95% CI: 0.672–0.823), demonstrating good predictive performance, which was comparable to that of the MELD score (AUC: 0.762, 95% CI: 0.681–0.827). Similarly, the AUCs for HDL-C and the MELD score for predicting 90-day TF mortality were 0.732 (95% CI: 0.668–0.796) and 0.729 (95% CI: 0.657–0.792), respectively. Upon internal validation, the AUCs for 28-day mortality were 0.827 (95% CI: 0.740–0.895) for HDL-C and 0.776 (95% CI: 0.641–0.871) for the MELD score. Moreover, HDL-C and the MELD score demonstrated similar AUC values (0.778 and 0.755, respectively) for predicting 90-day TF mortality. When compared to TC, LDL-C, and TG, HDL-C exhibited superior prognostic accuracy at both 28- and 90-day in both the training and validation cohorts (all *P*<0.05).

### Characteristics related to HDL-C levels

The characteristics of patients in the training cohort were analyzed based on their HDL-C quartiles (**Table 2**). The median HDL-C level was 0.20 mmol/L (interquartile range, 0.13 mmol/L–0.36 mmol/L). Compared with other subgroups, patients with HDL-C < 0.13 mmol/L had higher levels of TBIL, aspartate aminotransferase, INR, and MELD scores, whereas the levels of LDL-C, triglyceride (TG), TC, and albumin were lower. Notably, the incidence of HE was markedly elevated (41.1%) among patients with HDL-C < 0.13 mmol/L.

**Table 2:** Baseline characteristics of patients with HBV-ACLF based on quartiles of HDL-C.

Variables	HDL-C (< 0.13mmol/L)	HDL-C (0.13-0.20mmol/L)	HDL-C (0.20-0.36mmol/L)	HDL-C (> 0.36mmol/L)	*P* value
Age (years)	47.0 (43.0, 56.0)	46.0 (39.0, 53.0)	44.0 (37.0, 53.0)	48.5 (38.0, 56.0)	0.281
Male (%)	63 (27.3)	60 (26.0)	50 (21.6)	58 (25.1)	0.784
Cirrhosis (%)	68 (27.8)	61 (24.9)	52 (21.2)	64 (26.1)	0.535
Ascites (%)	57 (29.5)	46 (23.8)	44 (22.8)	46 (23.8)	0.340
GIB (%)					
HE (%)	30 (41.1)	13 (17.8)	11 (15.1)	19 (26.0)	0.020
ALT (IU/L)	190.3 (52.0, 516.5)	204.8 (52.7, 629.4)	115.5 (47.1, 58.8)	63.8 (30.1, 177.2)	0.065
AST (IU/L)	166.0 (90.2, 344.0)	177.8 (79.2, 547.0)	118.2 (54.9, 298.0)	73.7 (36.9, 185.0)	0.032
TBIL (µmol/L)	254.5 (180.5, 362.5)	228.0 (137.0, 325.7)	185.5 (110.1, 261.2)	117.7 (72.9, 207.5)	0.098
Albumin (g/L)	30.1 ± 7.0	30.7 ± 6.7	31.8 ± 7.7	34.9 ± 11.4	0.014
TC (mmol/L)	1.9 (1.5, 2.2)	2.6 (2.1, 3.0)	2.5 (1.7, 3.0)	2.8 (1.7, 3.7)	<0.001
TG (mmol/L)	0.7 (0.4, 1.1)	0.7 (0.5, 1.2)	0.6 (0.4, 1.0)	0.6 (0.5, 0.8)	0.001
LDL-C (mmol/L)	0.8 (0.5, 1.1)	1.2 (1.0, 1.7)	1.0 (0.6, 1.6)	1.4 (0.8, 2.0)	<0.001
WBC (×10^9^/L)	5.8 (3.9, 8.0)	6.1 (4.4, 7.5)	5.3 (4.0, 8.3)	4.7 (3.7, 6.6)	0.485
NLR	3.2 (2.0, 5.4)	2.8 (2.2, 4.3)	2.7 (1.4, 4.9)	2.5 (1.7, 4.1)	0.978
PLT (×10^9^/L)	88.4 (66.1, 123.2)	96.4 (56.4, 148.0)	99.0 (57.0, 142.0)	68.7 (42.4, 112.3)	0.164
INR	2.1 (1.7, 2.7)	2.0 (1.8, 2.4)	2.0 (1.7, 2.4)	1.9 (1.6, 2.3)	0.001
Cr (µmol/L)	67.0 (57.0, 84.0)	66.7 (55.0, 80.0)	68.2 (56.7, 79.5)	64.4 (53.8, 74.9)	0.177
Na (mmol/L)	136.0 (133.5, 138.1)	137.5 (134.5, 140.1)	136.7 (130.3, 140.0)	138.4 (133.4, 140.6)	0.009
MELD score	26.7 (23.0, 29.1)	24.6 (22.4, 27.8)	24.0 (19.8, 26.7)	22.0 (18.0, 24.9)	0.037
Mortality (%)	35 (51.5)	18 (26.5)	9 (13.2)	6 (8.8)	0.001

Data are presented as n (%), mean ± SD, or median (interquartile range). ALT, alanine aminotransferase; AST, aspartate aminotransferase; TBIL, total bilirubin; ALB,albumin; PLT, platelet count; TC, total cholesterol; TG, triglyceride; HBV, chronic hepatitis B; HDL-C, high-density lipoprotein cholesterol; LDL-C, low-density lipoprotein cholesterol; HE, hepatic encephalopathy; WBC, white blood cell; NLR, neutrophil-lymphocyte ratio; GIB, gastrointestinal bleeding; INR, international normalized ratio;Cr, creatinine; MELD,Model for end-stage liver disease; HBV-ACLF, hepatitis B virus related acute-on-chronic liver failure.

### HDL-C levels and 90-day TF mortality risk

**Figure 4:**
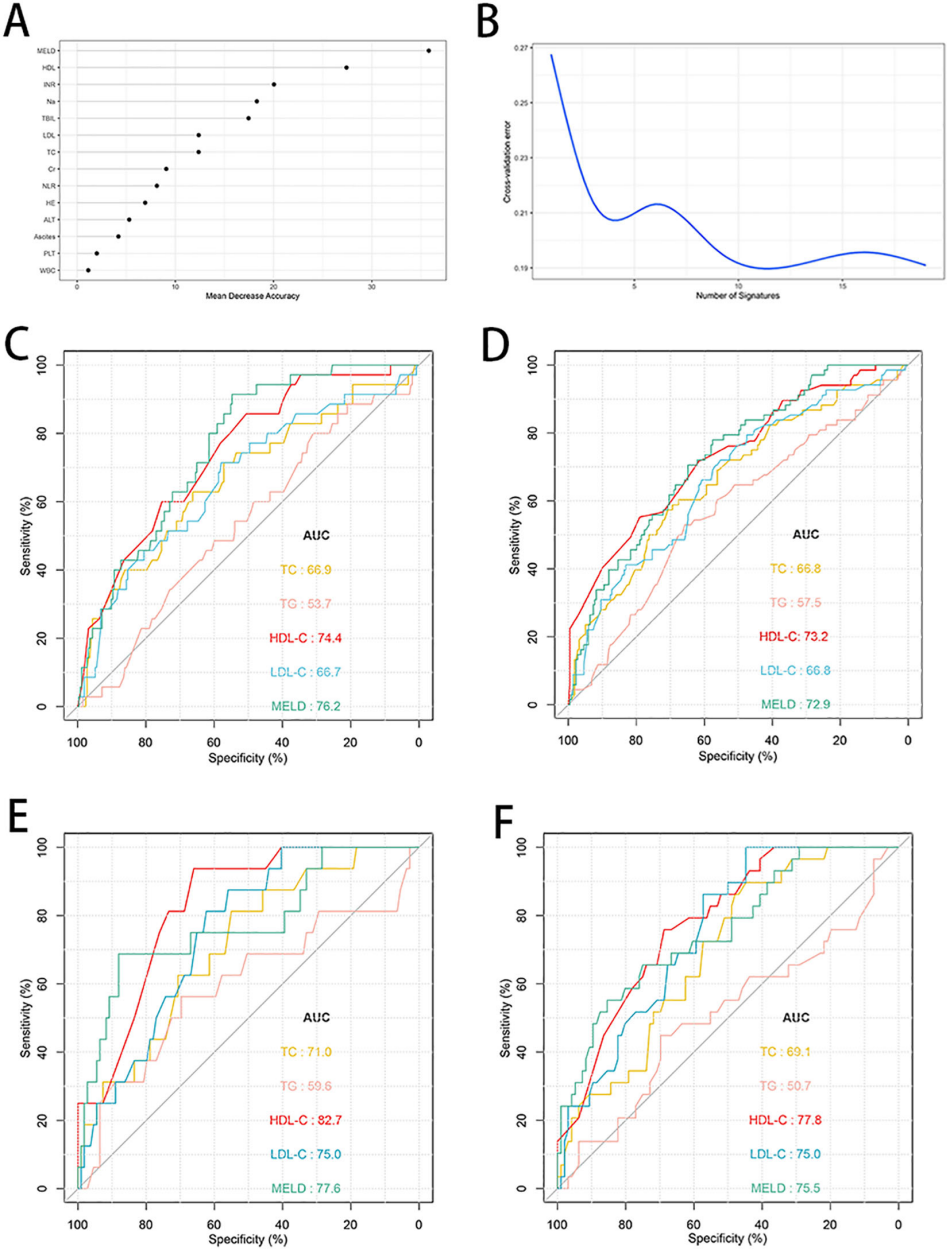
Random forest analysis and predictive ability of different indicators for 28- and 90-day TF mortality in patients with HBV-ACLF. **(A, B)** Random forest analysis with tenfold cross-verification indicated that HDL-C came in second only to MELD score in the training cohort (n=287). **(C, D)** ROC curves of HDL-C, LDL-C, TC, TG and MELD score predicting 28- and 90-day TF mortality in the training cohort (n=287). **(E, F)** ROC curves of HDL-C, LDL-C, TC, TG and MELD score predicting 28- and 90-day TF mortality in the validation cohort (n=125). TF, transplant-free; TC, total cholesterol; TG, triglyceride; HDL-C, high-density lipoprotein cholesterol; LDL-C, low-density lipoprotein cholesterol; MELD, Model for end-stage liver disease; ROC, receiver operating characteristic curve; HBV-ACLF, hepatitis B virus related acute-on-chronic liver failure.

The relationship between HDL-C levels and 90-day TF mortality exhibited a significant nonlinear L-shaped pattern, as revealed by the RCS analysis (non-linearity *P*<0.001). Lower HDL-C levels (< 0.20 mmol/L) were associated with an increased mortality risk, while higher levels were associated with a reduced mortality risk (**Figure 4**). Furthermore, the unadjusted and multivariable-adjusted HRs for 90-day TF mortality risk according to HDL-C quartiles were analyzed (**Table 3**). As a continuous variable, increased HDL-C levels were significantly related to a lower risk of 90-day TF mortality after adjusting for confounding factors (adjusted HR=0.02; 95% CI, 0.01–0.59; *P*=0.024). However, when assessed as a categorical variable, in the fully adjusted model, patients with HDL-C levels of 0.13–0.20 mmol/L had a higher risk of 90-day TF mortality, but this was not statistically significant (adjusted HR=2.33; 95% CI, 0.74–10.77; *P*=0.126). Regardless of multivariate adjustment, an HDL-C level < 0.13 mmol/L was significantly associated with an increased risk of 90-day TF mortality in patients with HBV-ACLF (all HR>4.0, all *P*<0.05), with HDL-C > 0.36 mmol/L serving as the reference group.

**Table 3:** Adjusted effects of HDL-C on the risk of 90-day transplant-free mortality in patients with HBV-ACLF.

	HR, 95% CI, *P* value Model I	HR, 95% CI, *P* value Model II	HR, 95% CI, *P* value Model III	HR, 95% CI, *P* value Model IV
All patients				
HDL-C (continuous)	0.02 (0.00,0.14) < 0.001	0.03 (0.00,0.24) < 0.001	0.02 (0.00,0.64) 0.026	0.02 (0.01,0.59) 0.024
HDL-C (categorical)				
> 0.36mmol/L	1	1	1	1
0.20-0.36mmol/L	1.89 (0.67,5.32) 0.225	2.00 (0.70,5.69) 0.194	1.61 (0.39,6.60) 0.503	1.35 (0.31,5.78) 0.686
0.13-0.20mmol/L	3.21 (1.27,8.10) 0.013	3.03 (1.17,7.84) 0.024	2.83 (0.60,9.02) 0.221	2.33 (0.74,10.77) 0.126
< 0.13mmol/L	6.41 (2.69,15.21) < 0.001	4.79 (1.93,14.93) 0.001	4.63 (1.09,15.84) 0.035	4.04 (1.35,11.85) 0.041

Model I was not adjusted. Model II adjusted for age, sex, cirrhosis, gastrointestinal bleeding, hepatic encephalopathy, and ascites. Model III adjusted for covariates in model II plus total bilirubin, neutrophil-lymphocyte ratio, and white blood cell count. Model IV adjusted for covariates in model III plus total cholesterol, prothrombin activity and international normalized ratio. CI, confidence interval; HDL-C, high-density lipoprotein cholesterol; HBV-ACLF, hepatitis B virus related acute-on-chronic liver failure.

### Subgroup analysis

**Figure 5:**
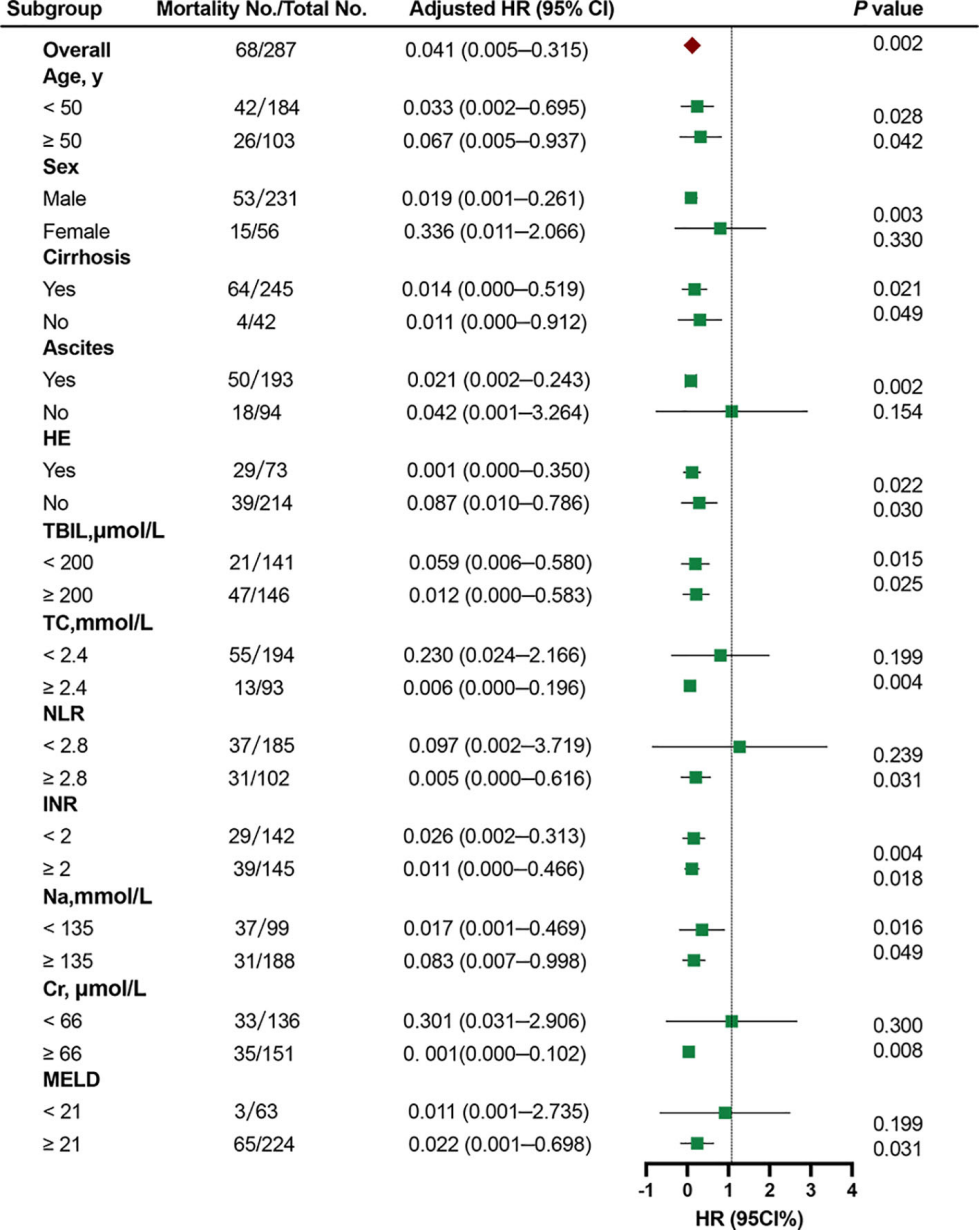
Cox proportional hazards analysis evaluating prognostic implication of HDL-C in differented subgroups in the training cohort. HR was adjusted for age, sex, ascites, hepatic encephalopathy, total bilirubin, total cholesterol, neutrophil-lymphocyte ratio, international normalized ratio, and creatinine in the multivariate model. HR, hazard ratio; CI, confdence interval; HDL-C, high-density lipoprotein cholesterol; TBIL, total bilirubin; TC, total cholesterol; NLR, neutrophil-lymphocyte ratio; INR, international normalized ratio; Cr, creatinine; MELD,Model for end-stage liver disease.

The relationship between the HDL-C and 90-day TF mortality was investigated in the training cohort through subgroup analysis according to age (≥ 50 years or < 50 years), sex (female or male), cirrhosis (yes or no), ascites (yes or no), HE (yes or no), TBIL (< 200 μmol/L or ≥ 200 μmol/L), TC (< 2.4 mmol/L or ≥ 2.4 mmol/L), NLR (< 2.8 or ≥ 2.8), INR (< 2 or ≥ 2), sodium (< 135 or ≥ 135 mmol/L), creatinine (Cr) (< 66 or ≥ 66 μmol/L), and MELD score (< 21 or ≥ 21). Continuous variables were classified according to cut-off values. Stratified analyses showed that patients with low HDL-C had worse outcomes in most subgroups (all adjusted HR<1.0). Although most of the results were statistically significant, the adjusted HR was more than 1.0 in female patients, patients without ascites, with TC < 2.4 mmol/L, NLR < 2.8, Cr < 66 μmol/L, and MELD score < 21 (**Figure 5**).

### Relationship between lipid-related biomarkers and inflammatory factor levels

**Figure 6:**
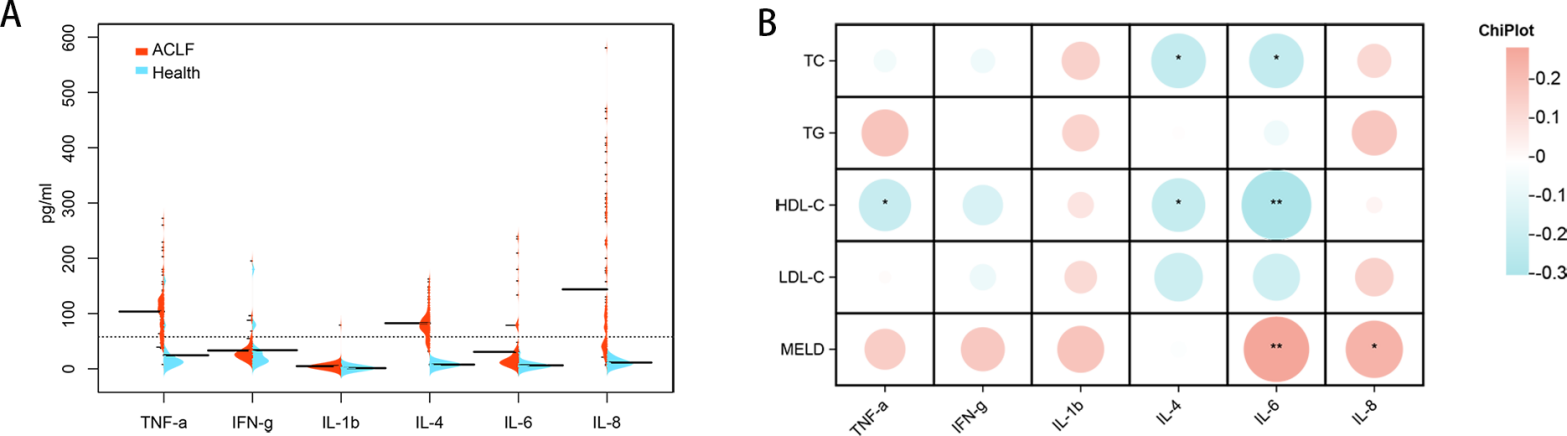
Inflammatory factors expression levels in healthy individuals and patients with HBV-ACLF and correlation analysis with lipid-related indicators. **(A)** Cytokine/chemokine expression levels in healthy individuals (n=20) and patients with HBV-ACLF (n=100). **(B)** Intestinal bacteria correlate with cytokines/chemokines levels in individuals with HBV-ACLF. A heat map was used to visually represent the color-coded Spearman’s correlations of these cytokines/chemokines, with red indicating a positive correlation and blue indicating a negative correlation (*P< 0.05,** *P*< 0.01). HDL-C, high-density lipoprotein cholesterol. HBV-ACLF, hepatitis B virus related acute-on-chronic liver failure.

In order to assess the potential correlation between lipid-related biomarkers and the inflammatory state, we measured plasma levels of inflammatory factors in both the healthy controls and patients with HBV-ACLF. The plasma levels of IFN-γ, IL-1β, IL-8, IL-6, IL-4, and TNF-α were found to be higher in patients with HBV-ACLF (**Figure 6A**). The MELD score was positively correlated with IFN-γ, IL-6, IL-1β, IL-8, and TNF-α, while HDL-C was negatively correlated with TNF-α, IL-6, and IL-4. Furthermore, TC showed a negative correlation with IL-6 and IL-4 (**Figure 6B**).

### Risk stratification for patients with HBV-ACLF

**Figure 7:**
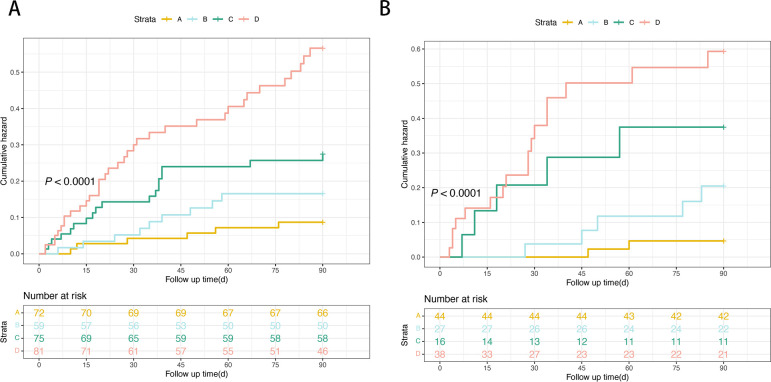
Risk stratification of 90-day TF mortality rate according to HDL-C quartile in the training **(A)** and the validation cohorts **(B)**. (Risk stratification: A: very low-risk group, HDL-C levels > 0.36mmol/L; B: low-risk group, HDL-C levels 0.20–0.36 mmol/L; C: intermediate-risk, HDL-C levels 0.13–0.20 mmol/L; D: high-risk, HDL-C levels < 0.13mmol/L; *P* < 0.0001 by log-rank test). HDL-C, high-density lipoprotein cholesterol.

Kaplan–Meier curves were plotted to illustrate the 90-day TF mortality based on the HDL-C quartiles. The 90-day TF mortality rates in the training cohort were 8.3%, 15.2%, 24.0%, and 43.2% in patients with HDL-C levels > 0.36 mmol/L, 0.20–0.36 mmol/L, 0.13–0.20 mmol/L, and < 0.13 mmol/L, respectively (*P*<0.0001; **Figure 7A**). Similarly, in the validation cohort, the 90-day TF mortality rates were 4.5%, 18.5%, 31.2%, and 44.7% in patients with HDL-C levels > 0.36 mmol/L, 0.20–0.36 mmol/L, 0.13–0.20 mmol/L, and < 0.13 mmol/L, respectively (*P*<0.0001; **Figure 7B**).

## Discussion

The pathogenesis of HBV-ACLF is complex and progresses rapidly, with a short-term fatality of approximately 50% (Moreau et al., 2021). Timely identification of prognostic risk factors is crucial for optimizing treatment and reducing mortality. Lipoprotein and lipid metabolism pathways are recognized as essential components of infection and immunity, involved in wound healing, bacterial lipopolysaccharide (LPS) binding, and clot formation (Barber et al., 2023). Our study identified an association between HDL-C levels and short-term outcomes in patients with HBV-ACLF, offering potential guidance for stratification due to its ease of calculation in clinical or bedside settings.

A recent study suggested that diminished HDL-C levels may represent a new prognostic biomarker for hepatocellular carcinoma progression (Crudele et al., 2022). To date, the utility of varying HDL-C levels and their association with the incidence of 90-day TF mortality rates in patients with HBV-ACLF has not been extensively investigated. Our current study revealed that nonsurvivors exhibited lower HDL-C levels than survivors. Cui et al. found that low HDL-C levels correlated with worse outcomes in patients with decompensated cirrhosis (Cui et al., 2022). Lipid levels are closely associated with nutritional status, sarcopenia, and hypoproteinemia, which are highly prevalent in patients with cirrhosis and ACLF (Kumar et al., 2022). We found a nonlinear relationship between HDL-C levels and the risk of 90-day TF mortality in patients with HBV-ACLF. Further analysis indicated that HDL-C < 0.13 mmol/L was related to an increased mortality risk (HR>4.0, *P*<0.05). The MELD score is a well-recognized prognostic indicator in patients with liver disease (Ruf et al., 2022). HDL-C demonstrated superior predictive ability than LDL-C, TG, and TC (all *P*<0.05) and was comparable to that of MELD score.

ACLF is commonly linked to a pronounced systemic inflammatory response (Zaccherini et al., 2020). Previous studies have indicated that HDL-related indicators and lipid mediators are associated with inflammatory markers (Trieb et al., 2016). HDL is the main lipid component of serum, which can bind cholesterol, phospholipids and other lipid components, and the synthesis is reduced in liver failure (Manka et al., 2014). Many lipid molecules can act as signaling molecules and are closely linked to inflammation. For example, arachidonic acid, as an omega-6 polyunsaturated fatty acid, is a precursor to certain pro-inflammatory eicosanoic acids (Schwarzkopf et al., 2019). A recent study demonstrated that HDL-C and apoA-I levels were negatively correlated with IL-6, white blood cell count, and C-reactive protein (Trieb et al., 2020). Reduced HDL-C levels were associated with an elevated risk and higher severity of infections. Studies have shown that HDL possesses the ability to downregulate the expression of pro-inflammatory cytokines induced by toll-like receptor (TLR) activation and directly neutralize the inflammatory activity of LPS, the main TLR4 ligand (Nardo et al., 2014). In our study, inflammatory factors were significantly elevated in patients with HBV-ACLF, and HDL-C was inversely correlated with IL-6 and TNF-α. Consistent with this, a prospective cohort study demonstrated that reduced HDL particle concentrations were linked to a heightened risk of sepsis-related hospitalizations and mortality. Genetic analyses suggested inflammation through IL-6 signaling as a potential mediating variable (Hamilton et al., 2023). The anti-inflammatory actions of HDL were identified by its ability to suppress TNF-α-induced expression of the vascular cell adhesion molecule-1 gene in endothelial cells *in vitro* (Jia et al., 2021). These results indicated that HDL-C plays an essential role in inhibiting endogenous inflammation, and reduced HDL-C levels are correlated with systemic inflammatory responses.

Accurate identification of high-risk patients is crucial for providing timely treatment and reducing ACLF-related mortality. A major finding of our study was that patients with HDL-C levels < 0.13 mmol/L exhibited a substantially higher 90-day TF mortality rate of 43.2%, whereas those with HDL-C levels ≥ 0.36 mmol/L had a markedly lower 90-day TF mortality rate of 8.3%. As noted earlier, clinicians could predict patient outcomes accurately based on HDL-C levels, enabling closer monitoring and timely implementation of therapeutic interventions. Therefore, a management strategy based on HDL-C may optimize medical resource allocation and improve the outcomes of patients with HBV-ACLF.

The current study provides a comprehensive analysis of the association between HDL-C levels and short-term clinical outcomes in patients with HBV-ACLF. Nevertheless, a few limitations should be acknowledged. First, while this study was prospective and formalized the predictive value of HDL-C through internal validation, it lacked the external validation from additional cohorts. Second, although this study examined HDL-C’s short-term prognostic value for patients with ACLF, future investigations are warranted to elucidate its potential value in predicting long-term clinical trajectories. Third, this study focused on the clinical characteristics of HDL-C changes. Ongoing research efforts in our cohort are focused on delineating the alterations in serum lipid metabolism that occur in patients with HBV-ACLF, with the aim of gaining deeper insights into the pathophysiological mechanisms driving this disease process.

## Conclusion

In summary, our analysis revealed that decreased HDL-C may contribute to the high mortality observed in patients with ACLF. Patients with HDL-C < 0.13 mmol/L had a worse prognosis. Monitoring serum HDL-C levels may aid clinicians in risk stratification and early management.

## Data Availability

The original contributions presented in the study are included in the article/**Supplementary Material**. Further inquiries can be directed to the corresponding authors.
