# Supplementary material for: High-density lipoprotein cholesterol as a prognostic marker for 90-day transplant-free mortality in hepatitis B virus-related acute-on-chronic liver failure
Source: Front Cell Infect Microbiol. 2025 Jan 22;14:1458818. doi: 10.3389/fcimb.2024.1458818 (PMC11794805; doi:10.3389/fcimb.2024.1458818)
Supplement: Supplementary file 1 [file DataSheet1.pdf]

ID	age	sex	time28	state28	time90	state90	ascites	HE
0001883240	44	1		0	55	1	1	1
0001873679	66	1		0	90	0	0	0
0001885508	47	2		0	90	0	1	0
0001900897	60	1		0	90	0	0	0
0001906854	55	1		0	90	0	0	0
0001906550	43	1		0	90	0	0	0
0001906369	43	1		0	35	1	1	1
0001908331	50	1		0	90	0	1	0
0001871448	55	2		0	90	0	0	0
0001908265	77	1		0	90	0	1	0
0000812307	38	1		0	90	0	1	0
0001921344	50	1		0	47	1	0	1
0002930888	40	1	18	1	18	1	0	1
0001900308	53	2		0	90	0	1	0
0001941418	46	1	15	1	15	1	0	1
0001943491	49	1		0	90	0	1	0
0001948996	53	1		0	90	0	1	0
0001956213	56	2		0	90	0	0	0
0000626208	38	1	24	1	24	1	1	1
0001841549	46	1		0	90	0	1	0
0001722164	58	1		0	90	0	1	0
0001846937	40	1		0	90	0	1	1
335935	36	2		0	90	0	1	1
548492	55	1		0	90	0	0	0
340503	44	2		0	90	0	1	0
329360	47	1		0	90	0	1	1
245349	50	1		0	90	0	0	1
234490	45	1		0	90	0	0	0
442275	58	1		1	35	1	0	1
371056	45	1		0	90	0	0	0
302722	58	1		0	90	0	1	0
353992	45	2		0	90	0	1	1
234641	30	1		0	90	0	0	1
234647	37	1		0	90	0	0	0
248907	51	2		0	90	0	1	0
234665	46	2		0	90	0	1	0
273046	27	2		0	90	0	0	1
234732	45	1		0	90	0	0	0
348323	41	1		0	90	0	0	1
236958	44	1	28	0	90	0	1	0

315030	33	1		0	90	0	1	0
358659	58	2		0	31	1	0	1
273111	31	1		0	90	0	1	0
340824	40	1	7	1	7	1	0	1
237064	60	2	28	0	90	0	1	0
245742	62	1		0	90	0	1	0
273152	38	1	28	0	90	0	1	0
237154	58	2		0	90	0	0	0
268098	36	1		0	90	0	1	0
437847	58	1		0	90	0	1	0
237184	65	2		0	90	0	0	0
372701	34	2		0	90	0	0	0
358662	34	1		0	90	0	1	0
273262	43	2	28	0	90	0	0	0
252654	27	1	28	0	90	0	0	0
273659	62	1		0	90	0	1	0
273768	49	1	28	0	90	0	1	0
273862	48	1	28	0	90	0	1	0
333859	47	1	28	0	50	1	1	0
375247	37	1		0	90	0	1	0
262309	67	2		0	90	0	1	0
349208	31	2		0	90	0	1	0
274335	28	1		0	90	0	1	0
274337	42	1		0	90	0	1	0
279007	48	1		0	90	0	0	0
344427	26	1		0	90	0	1	0
275021	30	1		0	90	0	0	1
347455	64	1	22	1	22	1	1	1
275117	30	1	28	0	90	0	0	0
242654	45	1		0	90	0	1	0
275152	28	1		0	35	1	1	0
399906	32	1		0	90	0	1	0
308880	49	2		0	90	0	1	0
242708	49	1		0	40	1	1	1
234893	46	2		0	90	0	0	0
244049	27	1		0	90	0	0	0
381293	30	1		0	90	0	1	0
243856	47	1		0	90	0	1	1
244460	43	1	28	0	90	0	0	0
326757	23	1		0	90	0	0	0
245141	46	1	28	0	90	0	1	0
334624	52	1	28	0	90	0	0	0
245455	60	1	28	0	90	0	0	0

302825	43	1	28	0	70	1	0	1
245790	41	1	28	1	28	1	1	0
302831	49	1	15	1	15	1	1	0
246100	65	1	28	0	90	0	1	0
246601	29	1		0	90	0	1	0
247079	37	1	21	1	21	1	0	0
246852	41	1	28	0	48	1	1	0
249030	46	1	28	0	90	0	1	0
247521	48	1	28	0	32	1	1	0
248621	38	1	28	0	90	0	1	0
404321	51	1	28	0	90	0	1	0
370356	31	1	28	0	90	0	1	1
248331	58	1	2	1	2	1	1	0
422715	45	1		0	90	0	1	0
248516	47	2	28	0	60	1	1	0
386349	32	2	28	0	90	0	0	0
248988	20	1	28	0	90	0	0	0
248866	53	1	28	0	90	0	1	0
376017	49	2	28	0	90	0	1	0
249520	54	2	28	0	90	0	1	0
315494	37	1	28	0	90	0	1	0
361158	69	1	6	1	6	1	1	0
249709	50	1	28	0	90	0	1	0
250222	67	1	28	0	90	0	0	1
249968	37	1	28	0	90	0	1	0
253956	56	1	28	0	90	0	1	0
286399	63	2		0	80	1	1	1
340880	40	1	19	1	19	1	1	0
365063	39	1	28	0	90	0	0	0
250465	53	1	28	0	90	0	1	0
373688	43	1	10	1	10	1	0	0
302902	62	1	7	0	90	0	1	0
250538	62	1	14	1	14	1	0	0
261376	26	1		0	90	0	0	1
292182	35	1	28	0	83	1	0	0
362190	31	1	28	0	90	0	0	0
469620	50	2		0	90	0	1	0
337965	33	1	28	0	90	0	1	1
438187	29	1	28	0	90	0	0	0
251993	36	1		0	90	0	0	0
281299	44	1	28	0	90	0	1	0
332559	43	1		0	90	0	1	0

302946	48	1	28	0	65	1	1	0
252181	35	1	28	0	90	0	0	1
251983	67	1	28	0	90	0	1	0
340617	33	2	28	0	56	1	0	0
252930	41	1	19	1	19	1	1	0
252892	35	1	28	0	90	0	0	1
325026	46	1	2	1	2	1	1	1
338499	53	1	28	0	90	0	1	0
281479	46	1		0	90	0	1	0
433935	44	1	28	0	90	0	1	0
252710	55	1	28	0	90	0	0	0
252655	37	1	28	0	90	0	1	0
254805	46	1	28	0	90	0	1	0
258715	44	1	28	0	90	0	0	0
255862	29	1	28	0	37	1	0	0
255415	50	2	13	1	13	1	0	1
258744	54	1	28	0	90	0	1	0
258855	38	1	28	0	90	0	0	0
399316	64	1	11	1	10	1	1	1
262305	53	1	5	1	5	1	1	1
259331	42	1	28	0	90	0	0	0
416722	34	1	28	0	90	0	0	0
260899	57	1	12	1	90	1	1	1
322059	57	1	28	0	90	0	0	0
260992	46	1	25	1	25	1	1	1
262123	37	1		0	90	0	1	0
386721	43	1	28	0	90	0	1	1
261802	27	2	28	0	90	0	0	0
296726	39	1	11	1	11	1	1	0
262311	51	2	8	1	8	1	1	1
308972	65	1	28	0	90	0	1	1
289439	37	1	28	0	90	0	0	0
262390	34	1	28	0	90	0	0	0
263571	45	1		0	90	0	1	1
263145	49	1		0	90	0	0	0
262921	50	2	19	1	19	1	1	1
262963	44	1		0	90	0	1	0
328898	52	1	28	0	90	0	1	1
333274	20	1	28	0	90	0	1	0
308984	22	1	28	0	90	0	1	1
344516	55	2	28	0	90	0	1	0
264542	46	1	10	1	10	1	1	0
264894	23	1	28	0	90	0	0	0

435532	54	1	28	0	90	0	1	1
263846	56	1	7	1	7	1	0	0
268223	38	1	28	0	90	0	0	0
265199	21	1	28	0	90	0	0	1
264477	55	2	28	0	90	0	1	0
264364	39	2	28	0	90	0	1	1
265067	42	1	28	0	86	1	1	0
266702	59	1	28	0	90	0	1	0
402105	50	2	8	1	8	1	1	1
264908	49	2	28	0	90	0	1	0
266129	40	1	28	0	90	0	1	1
265026	40	1	4	1	4	1	1	0
322088	43	1	28	0	84	1	0	0
265061	52	1	28	0	90	0	1	1
265184	44	2	28	0	90	0	0	0
336579	37	1		0	90	0	1	0
265329	39	1	28	0	90	0	1	0
265891	45	1		0	90	0	1	1
265390	42	1	28	0	90	0	1	0
421741	44	1	28	0	90	0	0	0
266267	44	1	2	1	2	1	1	0
266223	63	1	3	1	3	1	1	1
351414	46	1	28	0	90	0	1	1
269524	43	1		0	90	0	1	0
266926	44	2		0	90	0	0	0
329498	44	1		0	90	0	1	1
266611	56	1		0	90	0	1	0
424199	63	1		0	90	0	1	0
289491	46	1		0	90	0	1	0
266507	43	1		0	90	0	0	0
426905	42	1		0	90	0	0	0
439840	43	1		0	90	0	1	0
268317	53	1		0	90	0	1	0
430636	63	1		0	27	1	1	0
270003	42	1		1	78	1	1	0
387662	64	1		0	16	1	0	1
269357	42	1		1	66	1	1	0
269936	54	2		0	90	0	0	0
269717	55	1		0	90	0	1	1
271921	43	1		0	90	0	1	0
269820	53	1		0	90	0	1	0
271929	29	1		0	90	0	1	0

269919	67	2		0	90	0	1	0
439885	40	1		0	90	0	1	0
283620	71	2		0	12	1	1	0
272032	25	1		0	90	0	0	0
186441	40	1		0	38	1	1	0
188965	27	1		0	90	0	0	0
49431	62	1		0	90	0	1	0
149600	64	1		0	90	0	1	0
112927	50	1		0	90	0	1	0
160855	50	2		0	59	1	1	1
190146	52	2		0	90	0	1	0
191109	50	1		0	90	0	1	0
153291	55	2		0	90	0	1	0
190774	60	1		0	90	0	1	1
192100	43	1		0	5	1	1	0
192354	65	1		0	90	0	0	0
159610	56	1		0	76	1	1	0
193885	65	1		0	90	0	1	0
124812	40	1		0	90	0	1	0
194234	58	1		0	90	0	1	0
194282	45	1		0	90	0	1	1
170998	75	1		0	90	0	0	0
195760	35	2		0	90	0	1	0
196995	59	1		0	90	0	1	0
144670	54	1		0	90	0	1	0
183178	58	1		0	90	0	1	0
179318	34	1		0	90	0	0	0
183918	50	2		0	58	1	1	0
0000475785	59	1		0	90	0	1	0
85390	53	1		0	20	1	1	0
171206	40	1		0	90	0	0	0
184955	41	1		0	90	0	1	0
185053	48	2		0	90	0	0	0
102764	75	1		0	67	1	1	0
185287	37	1		0	90	0	1	0
184902	64	2		1	39	1	1	1
145512	53	1		0	90	0	1	0
155071	61	1		0	90	0	1	1
159214	40	1		0	90	0	1	1
160271	70	1		0	90	0	0	0
102225	66	1		0	28	1	1	0
146514	52	1		0	6	1	1	0
0000655797	56	2		0	90	0	1	1

124107	60	2		0	90	0	1	0
188657	66	2		0	90	0	1	0
179673	61	1		0	90	0	1	0
176468	50	2		0	90	0	1	0
146985	50	1		0	90	0	1	1
95772	43	1		0	90	0	1	0
89928	33	1		0	90	0	1	0
151191	53	1		0	90	0	1	1
191393	60	1		0	90	0	1	0
191910	43	1		0	90	0	1	0
88665	40	2		0	90	0	1	1
192213	60	1		0	90	0	1	0
193889	60	2		1	17	1	1	0
193871	50	1		0	90	0	1	0
125770	58	2		0	39	1	0	0
194946	40	1		0	90	0	1	1
195020	41	1		0	90	0	1	0
195851	35	1		0	90	0	1	0
196308	48	1		0	90	0	1	1
196682	18	1		0	90	0	0	1
107052	50	1		0	90	0	0	0
83847	55	2		0	39	1	1	1
141035	35	1		0	90	0	0	0
1325301963012	55	1		0	38	1	1	1
1101051963101	49	1		1	30	1	1	1
158702	67	1		0	90	0	0	0
113687	60	1		0	90	0	0	0
223014	45	1		0	90	0	0	1
222932	53	1		0	90	0	0	0
1102291980041	35	1		0	90	0	0	0
1303041958082	60	1		0	90	0	1	0
1325231966031	52	1		0	90	0	1	0
1326231972092	46	1		0	90	0	0	1
1102261967063	51	1		0	90	0	0	1
	33	1		0	90	0	0	0
0001876398	52	1		0	90	0	0	0
0001879101	36	1		0	90	0	0	0
0001897935	42	1		0	90	0	0	0
0001886174	51	1		0	90	0	1	0
0001904870	61	1		0	60	1	0	1
0001926797	50	1		0	90	0	1	0
0001813969	54	1		0	90	0	1	0

0001956336	40	1		0	57	1	1	1
0000938371	60	1		0	90	0	1	0
0001245049	65	2		0	90	0	0	1
0001805896	56	1		0	90	0	1	0
0000095471	49	1		0	34	1	1	1
0000678692	69	2		0	90	0	0	1
0000465400	51	1		1	40	1	1	1
0001954383	37	1		0	90	0	0	0
0000192923	57	1		0	90	0	1	0
0001964176	50	2		0	90	0	0	1
1	65	1		1	34	1	1	1
1	47	1		0	90	0	0	1
1	45	1		0	90	0	1	1
1265366	28	1		0	90	0	1	0
1295848	35	1		0	90	0	0	0
1305067	31	1		0	90	0	1	1
511329	65	1	28	0	90	0	1	0
1308730	53	1		0	90	0	0	0
1324828	68	1	11	1	11	1	0	1
1337656	53	2		0	90	0	1	0
995670	46	2	28	0	90	0	0	0
1347864	41	2		0	90	0	0	1
1353057	52	1		0	90	0	0	0
1029302	29	1		0	90	0	0	0
1059649	32	1	28	0	90	0	1	0
1076731	46	1		0	90	0	0	0
1091666	43	1		0	90	0	1	0
0001147996	33	1		0	90	0	1	0
0001147996	59	1	28	0	34	1	0	1
0001183665	30	1	28	0	90	0	1	0
1178221	55	1	28	0	90	0	1	0
1194550	51	1		0	90	0	0	0
476846	47	1	28	0	61	1	1	1
1216947	60	2	28	0	90	0	1	0
1239911	28	1		0	90	0	1	0
845286	28	1	20	1	20	1	1	0
845286	58	1		0	90	0	1	0
879105	31	1		0	90	0	0	0
879105	56	2		0	90	0	0	0
879105	43	1		0	90	0	1	0
892052	49	1	28	0	90	0	1	0
901814	30	1		0	90	0	1	0

1199229	52	2		0	90	0	1	0
962306	24	1		0	90	0	0	1
962306	33	1	28	0	90	0	1	0
961800	46	1	28	0	29	1	1	0
964698	38	1		0	90	0	1	0
964698	30	1		0	90	0	0	0
253002	46	1	28	0	90	0	1	0
253002	46	1		0	90	0	0	0
253002	40	1	28	0	90	0	0	0
982034	37	1		0	90	0	1	0
1014737	60	2		0	90	0	1	0
940395	30	1		0	7	1	1	0
940395	60	1	28	1	28	1	0	0
	48	1		0	90	0	0	0
	28	1		0	90	0	0	0
	24	1	28	0	90	0	0	0
	51	2	28	0	90	0	0	0
	55	2	28	0	85	1	1	0
	51	2	5	1	5	1	1	0
	70	1	21	1	21	1	1	1
	40	1	28	0	90	0	1	0
	36	1	28	0	90	0	0	0
	37	1	28	0	50	1	1	0
	31	1	28	0	90	0	1	0
	42	1	28	0	90	0	1	0
	42	1	28	0	90	0	1	0
	32	1	28	0	90	0	0	0
	44	1	28	0	90	0	1	1
	37	1	28	0	90	0	0	0
	62	1	28	0	90	0	1	0
	48	1	28	1	28	1	0	0
	39	1	28	0	90	0	1	1
	49	2	28	1	90	1	0	0
	46	1	28	0	77	1	1	0
	65	1	28	0	90	0	1	0
	52	1		1	4	1	1	0
	52	1	28	0	90	0	1	0
	36	2	28	0	90	0	1	1
	42	1		0	3	1	1	1
	50	1		0	90	0	1	0
	49	1	18	1	18	1	1	1
	41	1	28	0	47	1	1	1

	45	1	4	1	4	1	0	0
	69	2	7	1	30	1	1	0
	32	1	28	0	90	0	1	1
	34	1	28	0	90	0	1	1
	56	2	28	0	90	0	1	1
182884	1	40		1	8	1	1	0
183773	1	53		0	27	1	0	0
184817	2	50		0	90	0	0	0
185147	1	68		1	90	0	1	0
186181	1	50		0	90	0	1	0
186381	2	32		0	90	0	1	0
187118	1	53		0	90	0	0	0
187590	1	42		0	90	0	1	0
189040	1	45		1	16	1	1	0
189566	1	24		0	90	0	0	0
193168	1	29		0	90	0	1	0
125177	2	52		0	90	0	0	0
196432	1	25		0	90	0	0	0
197302	2	51		0	90	0	0	1
181918	1	55		0	90	0	1	0
164381	1	30		0	90	0	1	0
184731	2	53		0	90	0	0	0
181461	1	42		0	90	0	1	0
172172	1	39		0	90	0	0	0
186584	2	72		0	90	0	1	0
187945	1	40		0	90	0	1	0
173687	1	35		0	90	0	1	0
177237	1	60		0	90	0	1	0
112031	1	68		0	90	0	1	0
196199	1	50		0	90	0	1	0
196163	1	28		0	90	0	1	0
1102231981061	1	38		0	90	0	0	0
132821196810[illegible]	1	50		0	45	1	1	0

AST	ALT	TBIL	ALB	TC	TG	HDL	LDL	WBC	PLT
282.1	298	198.1	30.5	2.35	0.5	0.26	0.98	15.58	252
13.5	11.8	153.9	61.1	1.46	0.39	0.19	0.71	4.4	79
595.8	219.2	136.5	59.2	4.46	2	0.15	2.68	5.22	282
39.5	60.2	386	73.3	2.53	0.4	0.17	1.55	6.14	52
16.8	95.8	378.2	72.5	2.52	0.8	0.14	1.17	6.77	74
55.4	68.4	109.8	52.5	2.17	0.47	0.23	0.95	13.55	114
25	28	228.3	35.8	2.35	0.5	0.09	0.98	5.65	55
1012.4	547	254.7	66.3	2.7	1.44	0.14	1.36	8.3	155
42	86	219	24	2.02	0.53	0.32	0.64	2.28	31
1987.2	976.9	354.6	60.5	2.72	0.81	0.15	1.86	7.55	255
55.4	68.4	150	30	3.16	0.66	0.47	2.18	13	100
19.2	47.1	307.6	58.6	2.63	0.37	0.37	0.57	4.65	34
1577	1530	251	30	2.35	0.5	0.16	0.98	8.24	141
65	51	75	31	4	0.85	0.57	1.66	4.2	36
78.9	72	498	46	1.45	0.84	0.11	0.51	13.8	200
1469	765	137	34	3.48	1.75	0.18	1.96	3.5	164
62	75.4	230.4	35.5	2.72	0.66	0.55	1.58	4.57	89
219	198	226	28	5.5	1.95	1.2	3.1	6	230
980	989	124	30	2.7	0.8	0.28	1.36	6.2	173
401	108	178	31	3.36	1.11	0.22	2.4	4.5	142
15	32	163	31	1.54	0.48	0.11	0.75	4.24	81
2370	2444	142	41	2.88	1.5	0.31	1.11	8.57	141
14	58	138	29	2.88	1.5	0.31	1.11	7.45	90
156	53	202	25	2.9	0.55	0.24	1.85	9.46	99
102	54.9	170	28	4.3	1.56	0.27	2.75	8	100
137	341	228	32	2.87	2.11	0.13	1.49	7.2	120
475.7	402.7	153.2	58.1	2.46	1.28	0.13	1.22	5.75	121
1527.3	2346.7	174.5	56.9	2.18	0.98	0.1	1.24	4.01	88
106.3	152.5	651.2	57.6	1.8	0.5	0.15	0.76	8.93	105.1
23.1	43.9	86.8	62.5	1.8	0.44	0.18	0.77	5.12	77
2019	938	294.9	57.1	3.98	1.59	0.18	2.28	6.1	143
287.5	213.6	92.4	54.1	1.29	0.27	0.22	0.43	17.21	67
380.8	165.1	169.6	58.3	1.8	0.47	0.12	0.82	4.87	127
59.3	119.1	542	46.3	1.32	0.49	0.18	0.37	17.85	64
778.4	226.6	198.6	33.7	2.1	0.99	0.11	0.86	4.39	139.9
472	155.8	276.3	34.1	2.45	1.14	0.14	1.41	7.67	156
190.3	127.7	101.8	29	2.21	0.82	0.13	1.29	2.98	171.9
820.7	527.6	183.4	30.6	3.29	1.9	0.13	1.76	6.16	141
571.3	217.3	145.6	31.4	2.17	0.72	0.17	1.13	2.99	56.2
323.9	391.6	399.1	30.7	2.13	1.35	0.12	0.96	10.49	159

66	73.7	238.6	32.9	1.88	0.84	0.11	0.83	12.56	94
17.2	60.3	651.2	21.5	1.44	0.41	0.08	0.89	8.02	43
1018.4	570.9	250.2	33.3	1.65	1.25	0.2	0.79	8.32	121.4
49.2	91	399	31.5	1.5	0.9	0.12	0.69	2.23	68.3
674.6	674	335.9	30.9	2.71	1.49	0.14	1.16	2.71	59
41.7	110.8	219.2	32.5	1.49	0.45	0.11	0.67	2.77	65
115.5	176.1	431.5	22.3	1.73	0.68	0.2	0.41	4.81	70.3
845.1	500.9	313	32.6	2.07	1.55	0.12	0.77	2.12	70
48.7	96.9	242.8	34.3	2.32	0.5	0.16	1.4	2.95	75
60.7	58	155.2	25.3	2.6	1.4	0.12	1.47	2.82	73.8
14	48.7	254.3	35	2.2	0.43	0.5	0.8	2.97	35.9
29.7	61.9	112.1	28.7	2.31	0.67	0.49	1.05	3.14	65
128	89.1	501.1	31.2	3.28	0.68	0.33	1.95	8.73	157
2075.2	1445.9	288.5	28.4	2.3	0.55	0.12	1.17	3.64	85
231.6	132.2	218.1	40.3	3.55	0.8	1.5	1.4	4.19	89
504	1056	181.7	29.8	3.13	0.85	0.25	1.93	4.54	101.5
563.2	267.1	250.3	27.3	2.2	0.63	0.14	1.03	6.48	210.5
119.9	177.2	423.2	32	1.5	0.4	0.11	0.72	7.99	80
95.2	171.4	290.6	35.3	2.12	0.41	0.11	0.73	4.18	72.3
469.3	177.8	367.7	30.6	2.9	1.13	0.16	1.38	9.45	110.4
265	212.2	182.7	31.2	3.05	1.09	0.17	2.08	4.06	94
939.4	1089.4	272	34.5	1.77	0.47	0.14	1.14	6.36	133.4
433.4	103.5	321.5	26.1	1.89	0.4	0.11	1.07	9.9	135
67	58.2	56.2	28.1	1.79	0.4	0.44	0.92	1.69	76.4
381	319.5	463.2	30	2.99	2.84	0.15	1.3	10.07	164
171.2	172.3	455.6	41.5	3.65	0.71	0.22	2.12	5.05	109
330.7	234.8	236.3	31.1	2.77	0.95	0.16	1.28	6.7	76
728.9	232.1	290.6	31.9	2.08	0.73	0.11	1.04	4.04	65
772.4	343.4	325.7	33	2.6	1.4	0.14	1.2	6.33	163.5
1370.9	816.9	261.2	37	1.84	0.41	0.2	0.89	5.36	96
598.7	246.8	411.2	32.5	2.66	0.94	0.32	1.11	6.53	134
207.5	74.6	254.5	28.4	1.99	1.09	0.19	0.95	13.41	173
512.4	813.4	140.4	26.6	1.95	0.65	0.12	1.04	7.8	198
78.1	199.1	272.8	33.9	2.5	0.95	0.11	1.26	5.79	67
427.1	213.7	386.2	27.3	2.11	0.99	0.11	0.86	6.49	99
38.8	116.6	240.5	33.1	2.3	0.6	0.14	1.23	4.9	63
52.7	72	228	27.5	1.68	0.3	0.15	0.57	2.9	35.5
668.6	273.9	297.8	33.8	1.76	0.34	0.12	0.77	10.12	127
1127.9	814.4	153.5	25.5	3.4	1.8	0.2	1.86	4.69	42.4
520.9	301	258.7	40.7	2.3	1.23	0.12	1.33	4.59	74.2
2206.5	1236	226.5	31.3	3.64	1.37	0.18	1.98	8.73	189.1
51.3	62.6	252.1	25.1	2.2	0.8	0.11	1.3	2.43	43.6
134.8	100.5	108	24.3	3.55	1	0.7	2.35	6.7	40.2

193.73	306.1	159.6	30.3	2.4	1.78	0.12	0.98	7.2	162
182.9	347.2	366.7	31.1	1.57	0.87	0.11	0.6	6.72	118
1078.6	220.5	355.7	32.6	2.26	0.4	0.18	1.25	5.6	57.4
1512.1	1391.8	389.2	27.4	1.88	0.7	0.13	0.8	5.41	95
111.7	66.1	324.4	24	1.69	0.94	0.19	0.7	4.84	67.8
37	80	341.8	28.4	1.83	0.64	0.11	0.8	1.22	36
39	104.3	206.5	21.2	1.7	1.33	0.23	1	6.4	83.2
172.9	95.6	423.5	29.5	1.5	0.6	0.2	0.4	7.29	99
397	276.1	301	28.6	1.7	1.33	0.23	1	9.52	150.9
2366.3	940.9	212	33.6	3.1	0.7	0.33	1.5	5.46	132
275.1	611.6	365.3	36.7	4	0.5	1.2	2.4	3.97	88.4
170.3	133.6	319.2	33.2	5.1	1.3	0.26	3.6	15.25	104
2422.1	1892.3	226.1	38.4	1.04	0.5	0.07	0.5	7.49	171
787.2	314	241.4	31.1	2.4	1.25	0.12	1.2	5.99	163
27.8	53.4	240	29.9	1	0.33	0.08	0.3	2.68	28.2
318.7	627.8	108.4	33.5	3.7	0.5	1.4	2.1	2.3	64.1
110.1	69.5	473.3	29.8	3.7	0.5	1.4	2.1	4.42	66.4
70.7	75.2	428.3	40.4	1.5	0.33	0.15	0.5	7.49	102.3
47	63.3	149.4	34.8	1	0.3	0.16	0.55	3.45	79
603.1	347.1	233.3	35.1	2.4	0.7	0.18	1.2	3.33	70.6
133.4	199.7	139.1	28.6	3.2	0.74	0.16	1.34	7.3	94.4
1436.2	594.3	259.7	29.6	1.25	0.4	0.09	0.5	8.24	179
2152.4	1277.4	367.6	34.3	5.5	3.5	0.28	2.9	11.51	206
88.4	93.5	104.9	24.1	3.7	0.5	1.4	2.1	3.8	85.7
43.2	61.6	91.5	26.8	3.7	0.4	1.4	1.9	13.89	64
37.4	78.4	106.6	19.7	3.6	0.6	1.2	2.2	19.56	53
114.2	162.8	646.3	33.9	1.75	0.66	0.12	0.62	6.3	65.9
213.7	169.8	405.3	26.6	1.76	1.17	0.1	0.7	6.63	101.4
96.8	172.4	170.2	24.9	3	1	0.14	1.5	3.84	80.3
130.1	147.5	193	27.4	2	0.6	0.12	0.8	5.36	82.3
2779.8	1022.5	366.6	35.8	2.58	0.99	0.16	1.13	9.92	69.3
162.6	133.7	198.3	29.8	2.84	0.94	0.18	1.65	4.91	108
91.1	140.6	130.3	33.3	2.96	0.8	0.24	1.7	5.24	57.3
2484.8	972.7	305.5	33.1	3.6	0.6	1.2	2.2	6.46	233
151.1	113.8	377.6	25.8	1.85	0.5	0.1	0.6	5.46	132
66.9	83	355.9	30	2.4	1.2	0.2	1.1	9.52	150.9
304.8	155.9	113.4	32.6	3.6	1.8	0.14	2	7.59	148
2889.1	3500.7	152.2	37.1	2.5	1	0.2	1.33	5.96	113
139.2	108.5	327.4	31.7	3.4	1.2	0.15	1.99	14.63	96.4
282.4	214.6	139.9	31.4	3.26	1.2	0.18	1.96	6.37	201
233.3	151.3	347.4	32.8	1.5	0.5	0.3	0.83	5.02	76
1222.8	744.6	373.3	26.9	1.5	0.5	0.3	0.83	8.02	209

746.9	785.6	217.8	31.8	1.95	0.5	0.12	0.7	3.39	97
44.3	36.9	49.9	16	2.15	0.38	0.88	0.79	5.2	46.2
1923	1467	284.7	30.9	3.8	1.2	0.57	2.55	7.44	169
40.5	90.4	206.1	40.3	4.75	0.48	0.58	1.6	4	91.2
911.2	738	257.1	33	1.3	0.4	0.11	0.52	8.01	112.3
2515.4	1707.7	106.2	39.4	2.21	0.67	0.3	0.7	5.62	74.4
2838	1037.8	280	31.4	2.21	0.4	0.13	0.99	15.56	223
117.5	226.5	169.6	29.5	2	0.87	0.11	0.87	5.2	124
220	156.5	239.8	28.3	2.09	0.94	0.11	1.15	6.21	50
597.6	463.3	188.8	29.7	1.52	0.48	0.23	0.6	3.33	70.6
1680.2	1510.3	211.8	33.1	1.5	0.5	0.3	0.83	7.68	98.7
423.3	338.2	426.2	30.6	1.64	0.56	0.11	0.56	4.19	88
99.4	187	353.8	28.4	4.3	0.96	0.84	2.47	6.19	38.4
670.5	1002.5	227.2	30.6	3	1.5	0.15	1.76	5.69	97
2281.5	1064.6	271.2	40	1.8	0.76	0.15	0.6	6.13	76
32	68	431.7	27.7	1.25	0.48	0.09	0.33	6.44	147.4
126.4	158.4	181.7	23.2	1.9	0.59	0.51	0.7	6.73	40.4
2329.5	1182.2	192.2	38	1.9	0.59	0.51	0.7	6.13	76
444.7	292	386.8	30.2	1.96	0.63	0.13	0.91	6.28	218
372.9	232.7	620.4	38.2	1.25	0.48	0.09	0.33	1.48	121.7
116.9	105.1	172.3	28.8	4.2	1.27	1	2.6	7.68	98.7
339.7	183.5	116.4	41.3	2.82	1.9	0.14	1.33	8.47	139
733.5	257.6	434.6	29.2	1.91	0.31	0.16	1.01	5.96	110
89.2	107.7	120.4	22.4	2.4	0.76	0.55	1.3	5.41	198
33.4	114.5	177.7	17.7	1.36	0.37	0.08	0.45	7.89	27.4
51.6	72.1	245.8	36	2.4	0.76	0.55	1.3	4.61	84
152	116.6	285.4	23.8	1.96	0.86	0.11	0.78	3.29	95
887	682.3	85	37.7	3	0.75	0.66	1.5	6.53	90.4
251	144.3	559.9	26.9	2.7	2	0.15	1.18	4.15	67
56.6	129.3	254.5	20.8	1.3	0.2	0.13	0.62	3.48	100.6
1128.7	902.3	353.1	30.2	2.33	1.44	0.12	0.9	8.34	140
666.9	573.9	206.4	34.4	2.8	0.23	1.5	0.97	3.43	91.9
1120.2	684	154	33.7	2.52	0.6	0.15	1.4	7.37	115
76	103	502.4	33	2.1	0.4	0.17	0.63	6.98	99
71.6	182	259.9	33.4	3.8	0.8	0.78	1.5	4.44	58.1
61.9	113.5	521.4	26.4	1.36	0.54	0.07	0.22	4.53	72
495.2	1085.9	72.3	36.8	2.4	0.76	0.55	1.3	4.9	181
183.2	271.4	234.5	30.9	3.8	0.8	0.78	1.5	5.73	73
2779.5	1259	188.2	38.4	2.02	0.74	0.16	0.87	7.27	96.4
753.4	458.3	199.9	38.2	3	0.7	0.4	1.6	5.28	66
48.1	132.5	340.4	30.8	1.4	0.48	0.19	0.25	2.77	46.2
268.1	330.8	443.5	29.4	3.34	1.06	0.8	1.5	12.01	152
54	145.5	255.6	25	1.43	0.5	0.19	0.44	3.84	152.2

25.3	166	498.4	29.4	1.6	1.11	0.12	0.6	2.54	33
701.7	879.3	270.9	37.5	5.3	3.18	0.18	2.4	4.57	208.3
43.6	75.8	118.7	30.5	2.9	0.7	0.53	1.13	5.36	120
1331.4	842.1	246.5	35.9	2.15	0.9	0.11	0.94	5.69	97
593.5	577.7	136.1	29.2	3.8	0.8	0.78	1.5	3.97	155
612.7	270.3	306.5	27.9	2.19	0.48	0.15	1.06	6.23	56.3
522.8	251.9	231.1	28.1	1.14	0.5	0.07	0.36	6.67	86.4
224.8	222.7	178.3	34.2	3.03	0.6	0.28	1.63	4.97	125
162	131.5	544.1	32	1.6	0.68	0.12	0.85	13.51	76.5
463.6	281.8	321.3	27.4	2.23	1.23	0.11	0.88	15.07	88.4
36.1	39.1	56.8	25.6	1.7	0.66	0.4	0.65	1.9	100
110.9	166	204.5	26.7	2	0.8	0.16	0.5	8.76	43.4
2209.2	2133.5	287.9	34	1.87	1.44	0.1	0.49	5.33	122.4
17.8	49.7	242	29.9	1.7	0.66	0.4	0.65	2.1	57.4
42.4	49.4	199.1	25.6	1.4	0.4	0.13	0.38	6.07	85.7
588.7	214.5	125.6	31.2	1.85	0.54	0.26	0.86	4.53	225.5
729.4	522.6	266.5	33.7	2.91	0.98	0.18	1.6	7.95	140
476.6	277.3	142.2	27.4	1.7	0.66	0.4	0.65	9.18	95
92.7	114.6	246.1	19.4	0.77	0.27	0.15	0.16	6.04	43.4
159	128	179	33.4	1.7	0.66	0.4	0.65	10.82	115.4
695.9	730.7	322.4	34	4.72	2.12	0.15	3.39	11.33	181
65.9	97.6	577.9	33.9	3.33	0.45	0.17	2.33	8.8	41.4
52.7	89.4	233.1	29.8	3.6	1.7	0.12	2	9.14	103.2
59.3	93.4	95	29.5	3	0.3	0.23	2.7	5.29	120
62	75.4	230.4	35.5	3.02	0.6	0.75	1.3	4.19	252
57.5	118.2	190.6	31	2.46	0.57	0.27	1.8	4.19	57
306.5	287	417.7	29	2.21	0.92	0.16	1.37	5.75	48
46.3	85.5	144	24	1.57	0.53	0.07	1.25	9.15	80
29.4	79.2	155	37	2.68	0.45	0.14	1.58	5	84
21.8	48.8	309.7	21	2.7	0.8	0.18	0.95	11.7	37
826.3	822	138.8	31.6	2.61	0.7	0.16	1.07	5.81	135
35.5	23.3	221.8	31.1	2.43	0.4	0.33	1.12	3.78	34.4
99.5	64.2	138.1	24.2	1.76	0.35	0.3	0.98	5.17	45.4
41.1	144.9	209.9	25.7	3.5	1.6	0.13	2	2.81	52
370.3	170.4	334.3	30.6	1.5	0.38	0.11	0.66	7.16	106
45.8	11.4	139.7	28.3	0.25	0.16	0.09	0.32	12.31	63
69.9	34.2	248.9	22.6	1.26	0.47	0.09	0.5	12.02	92
90	164.8	207.9	24	6.34	1.91	0.4	4.7	3.9	213
117.5	43.2	102.6	32	2.44	0.5	0.9	1.06	3.94	46.4
15.4	96	170.3	40	3.3	0.9	0.76	1.84	5.91	191
38.5	17.9	215.2	30.7	2.6	0.32	0.24	1.28	3.46	29.4
1844.8	1251.4	124.4	37	4.68	1.2	1.5	2.16	8.57	169

32.5	29.6	219.5	26.1	1.76	0.28	0.18	0.9	4.95	48.4
74.4	195.4	233.8	23.2	2.65	0.21	0.1	1	9.07	104
99	49.6	116.7	30.7	3.8	1.45	0.4	2.24	7.34	96
109.4	250.1	222.4	40.1	3	3.17	0.3	1.27	4.28	172.4
86.4	32.2	116.5	26.9	2.8	0.5	0.14	1.1	19.15	200
319.2	136.5	370.2	37.5	3.8	1.95	0.18	1.9	6.45	151
77.1	53.5	100.9	18.4	1	0.18	0.5	0.56	11.29	32.4
370.6	462.9	358.3	26.4	1.86	0.48	0.12	0.79	4.32	20.1
55.5	23.8	128	21	0.92	0.35	0.4	0.32	2.1	69.4
223.4	136.1	410.6	34.9	1.26	0.47	0.09	0.5	6.71	53.4
200.4	46	283.1	31.5	2.67	1.8	0.1	1.4	10.32	196
32.1	113.2	110.1	39.2	5.3	6.6	0.19	2.89	9.61	196
80	40	105	29.9	2.9	0.5	0.3	1	8.75	84.4
26.2	59.1	143	27.9	1.4	0.33	0.12	0.5	8.35	99.4
248.6	157.9	158.7	20.8	2.7	1.6	0.11	1.4	2.52	63
1320.3	1033.9	267.3	34.2	2.47	1.5	0.1	0.97	3.84	67.4
29.2	131.2	100.7	26.2	1.76	0.17	0.6	0.85	6.5	119
303.5	97.1	190.4	34.2	3	1	0.17	2	3.89	100
24.8	43.5	165.3	19.4	2.4	0.35	0.14	1.22	3.94	50
39.9	62.7	150.8	30.4	3.5	0.5	0.4	2	7.08	132.9
31.5	32.3	155.3	35.4	3.3	0.55	0.86	1.46	7.21	42.4
656.6	435.6	132.4	34.4	2.8	0.97	0.29	1.5	4.74	137
387.7	473.1	131.1	30.9	1.3	0.27	0.12	0.43	17.21	67
60.9	26.4	113.1	27.9	4.68	0.8	0.68	1.9	5.98	54
32.6	19.3	172.6	23.9	2.2	0.33	0.12	0.7	3.6	33.4
61.9	28.6	95.3	44.7	2.9	0.76	0.2	1.45	3.76	63.4
49.3	108.1	49.3	31.9	4.45	0.8	0.1	2	4.93	60
13.2	14.7	95	24.1	1.84	0.32	0.28	1.29	2.29	15.4
9.7	12	19.5	26.8	2.95	0.5	0.26	1.75	5.31	46.4
76.8	62.4	16.3	33.4	1.52	0.33	0.16	1.1	4.46	51
16.7	30.6	53.5	25.2	3.2	0.44	0.28	1.36	3.64	67
35.1	21.6	46.7	30.8	2.82	0.7	0.16	1.38	3.52	64
611.8	403.8	61.6	34.5	2.88	1.35	0.16	1.9	6.49	80.9
58.2	77.2	68.6	24.3	2.9	0.65	0.15	1.65	2.62	35
68.3	42.5	76	25.9	4.24	0.97	0.36	1.08	3.7	29.4
45.2	26.4	56.6	27.4	1.85	0.67	0.15	1	3.1	57
15.1	28.1	27	26.8	2.66	0.8	0.9	1	4.73	103
10.6	12.8	61.6	27.8	1.77	0.4	0.5	0.89	2.45	66
104.4	349.2	91.3	24	1.77	0.4	0.5	0.89	6.31	38
16.2	9.3	22.2	28.3	2.3	0.8	0.5	1.46	4.14	29.4
33.8	20.7	35.7	30.5	2	0.6	0.66	1	1.45	15.2
19.8	34.4	16.4	24.5	1.4	0.54	0.31	0.71	4.05	33.2
62.7	19.8	81.6	30	2.7	0.34	0.97	0.98	3.91	7.4

25.9	27.7	17.4	33.1	2.15	0.5	0.9	1	3.77	37.4
206.3	141.1	32	17.1	2.15	0.55	0.3	1.37	3.41	44.4
52.4	18.8	78.3	30.5	1.95	0.37	0.4	1.2	3.35	53.4
40.6	44	28.1	33.9	4.57	0.9	0.16	3.2	9.58	39.4
8.3	13.1	13	24.3	1.92	0.16	0.25	1.16	7.1	148.4
50.3	22.6	24	21	2.53	0.47	0.15	1.23	4.88	234
24.1	18.2	62.1	21.3	2.37	0.3	0.7	1.4	2.99	22.4
14.3	26.3	63.1	36.7	2.53	0.4	0.7	1.5	2.16	36
17.9	21.8	46.2	20.7	4	0.77	0.5	1.36	10.58	42.4
77.9	94.4	27.2	25.9	3.53	0.67	0.1	1.67	2.5	50
25.4	16.6	14.8	33.2	1.61	0.7	0.6	0.55	0.9	17.2
21.3	17	27.2	28.8	1.64	0.9	0.12	1	2.58	36.4
19.4	35.6	24.1	27.4	2.28	0.56	0.14	1	2.14	41.4
56.1	44.2	85.3	31	5.3	0.6	0.6	3	7.79	68
14.1	48.8	30.3	29.2	3.3	0.37	0.2	1.5	4.59	86.4
95.9	30.6	78.8	32.6	2.45	0.28	0.27	1.3	6.65	245
26.8	9.3	45.7	32.1	1.54	0.3	0.83	0.6	2.2	21.4
116.8	95.7	22.8	36.4	1.54	0.3	0.83	0.6	2.64	49.9
23	19.3	8.3	24.3	1.6	0.46	0.65	0.8	3.89	42.4
430.6	128.2	8.3	50.9	1.6	0.46	0.65	0.8	6.18	207
19.7	14.9	12.5	27.7	1.5	0.54	0.66	0.86	6.55	117
34.8	13.2	23.9	20.1	2.4	0.5	0.16	1.2	7.09	231
910.6	592.2	70.2	44.2	3.55	0.7	0.17	2.1	3.99	159
17.1	32	41.8	22.9	1.64	0.3	0.17	1.2	6.81	89
20.7	18.7	8.1	23.5	3.12	1.4	0.08	1.3	6.55	92
219.9	128.5	468.4	34.4	1.75	1.13	0.11	0.7	4.42	66.4
42.1	131.7	317.1	26.8	3.5	1.93	0.14	2	7.16	105
345.3	238.7	124.4	34.8	3.2	0.34	0.15	1.27	3.6	113
1617.1	1492.3	128.5	35.9	2.5	0.78	0.17	1.2	5.65	149
46.9	100	185.5	30.6	2.2	0.57	0.23	0.88	3.47	63
28.1	36.9	148.9	27.6	5.1	0.56	0.7	2.5	3.54	48.4
47.1	28.8	546.6	35.8	2.86	0.7	0.25	1	12.64	53
2206.5	1236	226.5	31.3	3.65	1.37	0.48	1.98	8.92	195
941.2	737.9	223.9	38.2	2.7	2.95	0.14	1	5.52	104
106.2	21.7	34.5	65.2	61.7	3.16	0.66	2.18	2.97	90
107.6	481	263.6	231.3	51.4	2.48	0.63	1.59	7.93	138
104	684	211	188.7	32.8	2.48	0.63	1.59	5.98	151
97.4	511.7	442.4	296	60.8	2.24	0.78	0.84	5.59	126
104.8	134.2	80.2	116.1	68	1.74	0.62	1.07	8.3	110
103.9	1340.1	1017.5	228.3	65.6	2.49	1.23	1.16	4.63	138
100	55.4	68.4	170	30	4	0.85	1.66	10	90
108	62	75.4	230.4	35.5	2.72	0.66	1.58	4.57	252

94	940	983.2	494	30.1	3.2	0.97	1.49	5.22	92
102	87	103.4	256.1	30	4	0.85	1.66	4.83	78
107	1473.2	1059.1	179	38.6	4	0.85	1.66	4.6	89
100	38	192	182	73	2.72	0.66	1.58	5.2	93
109	387	429	397	24	2	0.93	0.77	3.05	35
96	33	34	86	33	3.24	0.3	1.58	4.91	48
104	102	54.9	166	30.4	2	0.93	0.77	2.73	123
91	84	119	84	28	2.7	0.5	1.8	10.3	89
103	167	116	187	35	3.9	1.6	2.47	4.2	97
104	374	238	177	36	4.3	1.56	2.75	5.3	196
98.4	951.1	443	325.9	57.1	1.62	0.39	0.7	4.86	80
93.5	528.8	344.3	404.9	59.7	3.2	1.7	1.66	11.19	150
105	340.3	76.4	48.2	69.8	3.9	1.6	2.47	6.7	191
102.7	317.9	282.2	312.2	29.5	4.8	1.28	3.74	4.75	94.4
104.4	504.6	316.4	198.6	32.8	3.9	1.6	2.47	5.83	59
101.9	497.6	467.5	111.8	31.9	2.51	1.18	1.3	3.49	111
108	312.5	575.5	295.2	23.1	1.71	0.5	0.79	2.23	119
106.6	28	75.3	44.1	26.6	2.09	0.54	0.99	1.7	49
95.7	1042.2	1628.8	433.7	30	2.28	0.63	1.15	4.54	99
97.5	16	37.4	118.1	36.6	2.87	0.44	1.58	1.41	88
107.1	163	63.6	252.3	27.6	1.94	1.2	0.85	3.71	136
107	184.9	215.3	444.8	32.2	2.12	0.41	0.73	3.71	158.5
98.6	43.6	55.5	309.1	28.3	1.68	0.43	0.57	2.08	59.6
101.4	1286.9	909.1	296.6	33.3	3.46	1.5	2.03	7.3	170
106.4	2009.6	771.5	140.2	37.4	4.01	0.84	2.04	4.95	125
100.1	459.9	226.7	328.4	30.1	1.91	0.69	0.9	6.9	123
106.2	182.3	42.7	351.9	29.1	4.3	1.77	2.58	5.1	121.7
104	86.4	124.1	181.2	34.9	2.87	0.44	1.58	5.48	50
93.8	24.1	61.9	374.1	40.2	2.12	0.41	0.73	6.63	75
93.9	233.6	76.6	343.8	24.4	1.04	0.4	0.15	7.4	69.4
102.8	133.9	180	524.1	35.3	1.72	0.58	0.53	6.79	83.4
107.1	65.3	126.7	185.6	24.8	2.45	0.42	1.26	8.59	123.5
107.1	927.3	411.2	374.1	40.6	2.4	1.78	0.98	5.22	136.2
105.6	212.3	133.1	383.6	28.7	2.52	0.7	1.46	5.9	115
102.9	334.3	260.3	190.2	34.5	2.39	0.82	1.45	6.77	157.6
101.7	73.3	75.9	411.2	31.9	1.79	0.45	0.98	2.21	57
101.1	54.1	77.5	144.4	26.1	2.87	0.44	1.58	4.67	71.5
104.6	1738	1025.6	177.1	31.5	2.22	1.63	1.16	10.43	245
104	58	88.4	152.2	29.5	1.27	0.31	0.68	2.85	60
107.5	322.1	186.4	107.5	30.6	3.59	1.55	2.36	4.42	155.2
102.4	220.3	185.2	358	29.5	2.24	0.83	0.98	9.18	105.4
103.3	114.2	217.9	435.7	38.1	2.27	0.6	0.9	4.27	76

104.6	990.6	982.3	133.4	36.9	3.54	0.71	1.68	3.61	75.4
106.5	74.9	136.8	302.2	30.2	2.49	1.76	1.29	2.5	107.4
99.2	1123.6	343.8	109.8	37	1.94	0.25	0.94	4.71	90
95.8	54.6	115.3	377.8	31.6	2.29	1.46	1.21	5.88	111
88.2	1266.6	929.4	300.7	28.1	1.7	1.57	0.8	9.43	168
102	862	490.6	547.3	37.8	6.03	6.67	2.79	8.45	128
99.8	343.2	232.4	430.6	34.6	2.09	0.9	1.09	6.77	154.1
103	28.9	43	119.1	36.7	1.87	0.49	0.71	3.66	39
104.4	243.3	315.1	88	26.9	3.5	0.45	1.77	2.47	49.4
105	466.8	213.7	127.5	36.1	2.38	0.81	0.89	4.82	66.4
104.7	131.9	95.2	351.7	33	1.88	1.11	0.72	3.92	44.4
99.1	424	772.3	272.8	40	3.09	1.2	1.38	6.19	98.4
99	1455.8	859.1	287.4	30.2	2.4	1.78	0.98	5.63	121.7
100	127.4	306.4	425.2	19.5	2.87	0.44	1.58	11.33	119
104.3	1245.2	827.9	255.8	30.3	1.98	0.86	0.91	9.8	199
100.2	2580.6	1142.3	296.2	36.2	2.21	1.3	0.84	4.26	99.3
102.2	1045.8	1046.3	179.2	37.9	2.04	0.53	1.08	6.07	98.3
97.7	39.1	121	250.5	27.2	1.31	0.74	0.47	5.19	100.6
91.2	59.6	128.2	341.8	24.6	0.99	0.39	0.26	12.51	62.4
105.1	348.7	212.4	341.8	32.5	2.05	1	0.65	7.91	112.9
97	48.1	80.4	84.3	26.9	1.55	0.55	0.9	4.3	56.7
105	1539	1344.2	85.6	37.9	3.5	1	1.67	5.38	124.9
103	521.1	433.8	321.4	33.1	2	1.44	0.68	9.04	101.4
99	64.3	79.1	367.3	20.1	0.92	0.47	0.2	15.7	59
101	98.9	83.9	105.4	32.8	2.46	0.82	1.15	5.6	57.4
97	67	88.3	298.1	34.2	1.98	0.4	0.84	3.57	99.4
108	1132.6	930.3	137	38.5	2.6	0.6	1.06	4.06	119
102	125.7	232.4	262.3	38.9	1.3	0.4	0.54	12.23	165.7
100	702.6	659.9	217.2	37.3	4.35	1.4	2	9.08	113
104	175.9	82.4	397.2	24	1.8	0.5	0.4	10.96	71.4
106	327.1	179.3	141	28.1	2.4	1.8	0.8	2.36	47.6
108	680.8	650.6	110.1	32.5	3.55	1.2	1.86	8.01	112.3
101	325.4	197.4	215.8	31	3.6	2.4	1.5	7.65	88.4
95	799.2	341.6	192.6	41.3	2.6	0.85	1.52	11.84	237
105	124.6	110.9	172	27.9	3.6	0.6	2.2	7.04	66.3
104	180.3	218.5	444.1	27.3	1.9	0.7	0.9	2.31	119
107	969.9	804.5	171	32.8	2.3	0.5	1.1	5.47	46.4
102	89	241.8	112.5	38.8	2.5	0.6	1.04	7.12	68.4
96	120	536.6	266.2	34.8	1.27	0.65	0.38	3.63	68.3
104	137.2	207.3	333.4	31.1	2.74	1.8	1.24	4.68	43
105	93.2	55.5	602.2	31.7	2	0.5	0.84	4.92	33.4
82	38.6	59.2	163.8	23.6	2.5	0.4	1.2	3.9	69

96	120.7	117.2	548.3	25.2	1.33	0.57	0.4	7	70
86	331.8	326.4	237.7	23.2	0.85	0.4	0.17	6.45	47.4
103	67.7	180.7	547.3	32	1.65	0.8	0.57	7.65	139.3
106	297.5	341.2	90.4	26	3.02	0.6	1.3	7.29	69.4
105	332.7	299.7	206	24.2	2.83	0.78	1.6	4.93	98.7
86.3	65.3	43.3	170.3	26.3	2.56	0.36	1.04	3.18	54
103	747.2	1382.5	148.3	38.1	2.7	1.1	1.13	6.23	159
99	908.1	605.9	160.6	31.2	1.86	0.65	0.7	7.96	78
98	1535.6	841.8	164	34.1	2.87	0.5	1.4	6.33	104.4
109	1013.4	1168.4	212	33.4	2.3	0.7	0.86	3.37	98
111	82	111.7	213.7	30.6	1.65	0.61	0.7	4.2	73.4
105	245.5	3029.5	218.3	40.9	3.65	1.41	2.11	4.84	161
110	123.5	101.3	106.7	24.2	2.65	1	0.62	23.27	59.5
91	26.5	13.6	242.6	28.5	1.1	0.3	0.48	19.35	72
102	2061.9	506.4	178.5	40	3.87	2.14	1.9	5.18	127
100	1257.1	1928.7	236.3	31.2	1.65	0.4	0.7	6.38	97
102	646.4	484	223	30.6	7.76	2.8	6.15	3.61	190
102	2384.2	3086.9	109.1	39.7	3.2	1.8	1.44	6.53	131
110	22.4	18	127.2	31.4	1.46	0.26	0.5	2.23	45.4
97	48.5	25.3	62.1	29.8	3.8	0.6	1.6	5.24	54
98	38.1	14.7	51.4	28.7	3.46	0.73	2.25	4.87	111
101	37.4	39.4	21.7	44.8	1.86	0.3	1.36	2.93	41.4
114	25.9	41.6	19.6	25.3	2	0.32	0.69	5.95	53.5
103	25.6	36.3	85.8	32.8	5	8.4	1.22	4.71	61.7
100	41.4	49.4	56.6	39.7	3.84	0.44	2.3	4.51	66
108	16.1	12.6	31.1	30.2	2.2	0.14	1.4	1.55	26.4
108	50	36	52.7	26.8	3.9	0.97	1.84	4.68	83
99	30	24.3	34.9	30.6	1.66	0.9	1	11.79	38.4
108	72.5	22.7	40.9	37.4	2.4	0.5	1.6	3.44	58.4
104	37.7	20.5	52.2	27.8	3.3	0.6	1.84	3.91	76
104	35	34.7	16.3	31.4	2.7	0.6	1.6	4.22	66
	22	58.6	34.5	25.1	4.88	1.37	3.3	4.32	49.8
	638.4	422.6	337.8	30	1.65	0.48	0.32	10.85	156

INR	Cr	K	Na
2.34	58	135.5	106.2
2.32	154.5	3.35	131.6
1.58	41.9	3.62	139.5
2.39	93.5	4.53	133
2.81	57.8	3.4	137.3
2.48	127.2	5.84	123.3
2.74	70	4.18	136.8
2.32	64.8	3.86	141.2
2.51	78	4.22	130
2.93	91.9	3.61	143.4
1.6	60		135
3.32	48.2	3.61	136.9
1.8	78	3.68	137
1.98	52	3.9	142
2.28	76	3.1	137.8
1.6	77	3.67	142
1.7	58.6		137.3
1.5	59	4.1	140
2.5	61.9	4.54	136.3
1.5	76	3.86	135.6
2	84	3.4	136
1.9	77	3.38	140
2.14	56	3.8	137.6
2.3	66.4	3.8	144
2	78		140
2	91	4.88	139
1.66	60.6	3.64	141.8
3.68	63.4	4.07	135.5
2.78	48.3	3.23	129
2.01	67	4.74	133.8
2.31	82.3	3.61	143.9
3.09	76.2	4.1	136.1
2.85	53	3.49	138.2
3.41	232.4	3.73	128.9
2.25	49.3	4.15	138.6
2.14	91.9	4.54	140.1
2.16	51.6	3.16	139.9
1.69	67	2.26	137.6
2.31	87.6	3.51	144.8
3.15	63.5	3.81	136.4

2.56	67.6	3.33	132.2
2.78	73.2	3.74	133.4
1.94	66	3.52	141.8
2.14	48.5	4.08	129.8
2	47	2.8	138.2
2.12	56.1	3.79	143.6
2.92	132.8	2.72	139.3
1.56	45	3.64	139.4
2.2	63.5	3.45	133.5
2.06	65.7	4.01	141.6
3.26	50.2	4.47	140.5
2.93	52.8	4.31	141.5
2.61	59.1	3.95	133.2
2.98	48	3.57	134.9
2.88	52.4	4.38	138.9
2.2	79.5	4.81	140.5
2.55	90.2	3.2	143.6
2.26	79.5	4.47	136.3
2.5	56.9	3.91	132.8
2.13	49	3.52	139.2
1.81	54	3.58	143.9
3.03	50.8	3.53	134.8
4.04	75.1	3.28	138.5
2.64	80.9	3.66	141.1
1.67	66.4	3.37	136.7
2.08	71.5	4.18	141.4
1.87	64	3.59	142.4
1.91	91.6	4.38	141.4
1.91	80.7	3.98	139.6
2.69	60	3.39	141.6
1.73	60.5	4.02	142.6
1.94	64.9	4.32	139.8
2.11	45.4	3.13	141.8
2.03	84.9	4.02	137.7
2.28	71.2	3.29	139.5
2.58	84.3	3.68	138.2
3.34	75	3.85	136.9
3.07	102.2	5.2	127.6
2.04	68.2	3.74	138.3
1.63	57	4.42	137.6
2.05	86	3.78	143.3
1.73	66	3.24	137.3
1.8	150.5	4.73	129.2

1.59	64	4.42	133.9
2.03	91.5	3.18	132.3
1.83	48.8	4.4	136.4
2.13	61.7	3.24	134.5
2.27	87.6	3.66	135.7
1.84	117	4.19	136.4
1.7	80		135
1.77	46.9	2.46	136.4
1.88	69.3	3.7	127.7
1.72	70	3.53	141.7
1.29	47.9	2.66	130.9
1.79	50.2	3.58	137.5
6.66	111.6	6.53	135.5
3.08	41.7	4.17	133.5
1.8	57	2.86	127.9
1.96	57	3.85	135
2.03	57	4.41	126.1
2.02	51	4.11	135.3
1.86	39.7	4.92	135.7
1.83	51	3.52	134.5
1.96	85.5	4.17	132.6
2.69	122.8	4.1	136.2
1.99	88.2	4.37	137
1.95	61	3.67	132.6
2.33	79	4.36	136.5
2.27	169.4	4.08	128.1
2.12	111	5.33	129.9
1.65	84.1	3.93	135
1.93	60.8	4.14	136.6
1.96	85.5	3.78	138.9
2.24	65	3.97	140.1
2.23	65.6	4.22	144.2
1.69	101	4.74	125
3.07	81.8	3.81	143.3
1.55	75	3.86	131.7
1.88	84	3.7	137.3
1.99	70	4.3	135.6
4.17	84.3	4.77	137.6
1.79	73.3	4.18	134.5
2.17	63.4	4.05	133.2
2.2	66.2	3.43	133.5
2.44	68.5	3.31	142

2.33	94.5	4.97	133
2.7	115	3.44	140
1.78	85	4.11	135.8
3.03	56	4.17	128.8
2.93	59	2.8	140
1.92	48.4	3.63	127.3
6.93	84.8	4.68	131.5
3	62.5	3.01	139
2.63	87.6	4.39	137.7
1.83	56	3.89	138
1.66	68	4.72	134.2
1.91	42.3	3.94	137.4
1.74	74.9	4.79	133.4
1.74	80.8	3.33	140.5
2.57	74.9	4.2	133.9
2.14	68.8	2.29	127.2
1.79	54.7	4.34	133.2
2.57	74.9	4.24	140.8
3.22	72.4	3.63	122.3
2.4	54	3.89	132.9
1.66	68	4.13	136.8
2.16	80.7	4.6	143.4
2.26	52.6	3.64	135.8
2.13	61.7	5.72	125
2.76	103.4	3.75	127.2
2.05	75.2	3.93	138.7
2.02	58.6	3.57	133.2
2.05	60.2	3.46	137.7
2.15	87.7	2.97	137.9
3.73	55	3.8	136.4
1.75	50.1	3.14	134.6
2.68	65	3.86	143.3
1.63	61	3.59	139.1
3.55	71.7	3.16	140.4
2.3	76.9	4	139.6
1.92	30.2	4.79	127
1.75	69.9	4	139.6
1.84	68.3	4.02	138.9
1.8	56	3.12	140
3.13	55.5	4.35	140.7
2.53	63.3	3.85	136
1.64	66	4.2	133.8
1.98	44	3.04	136.7

3.75	91.6	3.97	141.7
2.16	75	3.07	133.8
1.72	57	3.73	134.4
1.74	80.8	3.27	138.1
2.26	57.5	3.96	142
2.35	50.5	3.6	134.5
1.72	59	3.89	134.6
1.98	75.9	4.59	134.6
2.33	45.6	4.68	131.3
3.97	106.5	4.03	135.2
1.81	80.2	3.88	144
2.05	80.1	4.02	123.2
2.02	65.1	4.27	139
1.81	43	3.67	137.1
1.83	48	4.14	134.3
2.8	87.5	4.11	140.9
2.95	84.3	3.33	137.5
2.12	79.6	4.47	139.4
2.16	566.5	5.17	131.5
1.8	74	4.87	130.8
1.85	236.8	3.19	123.7
3.25	210.2	4.47	133.3
2.06	64	3.97	132.9
1.66	78		129
2.3	66	3.56	139.3
1.78	66	3.56	139.3
2.67	55	4.45	133
2.55	157.9	4.21	134.5
2.17	66	4.4	136
3.2	56	3.3	132
1.71	75.3	4.27	138.3
2.63	39.3	4.24	112.2
1.75	61	3.55	132.4
1.23	76	3.26	136.7
1.77	79.1	4.77	130.7
2.58	345.6	6.31	137.4
1.5	198.1	5.72	134.6
1.09	41	3.1	134.8
1.35	69.4	3.63	132.6
1.34	58	3.26	138.4
2.71	112	4.85	119.3
2.46	77.8	5.75	138.4

2.31	71.2	3.62	128.5
2.16	59.4	3.15	132.1
3	6.32	4.42	140
1.11	69.3	4.18	136.7
2.41	49.6	4.47	128.7
1.46	75.4	3.51	139.1
2.79	65.9	6.19	125.9
2.7	73.5	3.58	135.6
1.84	62.2	3.93	122.9
1.44	60.2	4.04	134.2
1.45	45.7	3.34	133.5
1.34	41.8	4.3	132.1
1.39	56	3.68	135.2
3.19	70.6	4.3	124.3
1.44	33	2.91	133.4
1.83	58.2	4.17	137.2
1.59	61.5	3.21	135.2
1.92	54.5	3.95	142.5
2.44	65	3.95	137.3
2.04	67	3.32	141.8
2.16	67	5.06	136.6
1.09	57	3.69	139.9
3.09	76.2	4.1	136.1
1.96	63.5	3.6	137.4
1.63	67.5	3.89	134.7
1.79	72.6	4.73	134.8
1.56	67.9	3.67	138.8
2.36	55.7	3.27	130.3
1.56	61.6	4.17	140.1
1.94	66.7	3.89	144.6
1.98	72.5	3.56	130.3
1.88	74.3	4.38	141.8
1.69	54	4.01	139.5
1.73	73	4.13	139.5
2.06	53.3	3.57	141.6
2.44	3.9	3.32	140.6
1.73	85.2	4.18	139.9
1.69	66	3.22	135.1
1.57	67	3.02	136.2
2.11	63.8	4.28	141.2
1.56	55.8	3.86	135.3
2.06	97	4.2	140.6
1.8	75	4.71	126.2

1.54	41.1	4.35	140.6
1.8	65.3	3.78	137.3
1.56	73.5	4.1	138
1.8	114.4	3.82	138.4
1.6	63	4.37	144.1
1.74	50.9	4.38	139.7
1.73	90.6	4.04	142.4
1.82	61	4.01	138.3
1.57	99	3.91	140.5
1.57	74	3.98	141.7
1.73	43.1	3.74	139.8
1.96	61.4	4.37	136.9
1.86	63	3.48	139.7
1.64	60.7	4.31	133.5
1.9	44.1	3.84	142.8
1.81	28.3	3.74	136.5
1.93	45.1	2.71	138.7
1.54	68	3.79	143.2
1.77	74.2	4.31	147.2
3	83.7	4	138.5
1.56	25.1	3.95	143.8
1.72	32.4	4.03	127.8
1.58	76	3.75	136
2.1	57.6	3.08	135.8
1.78	65.9	4.08	140
1.78	61.2	4.16	137.3
1.69	67.6	4	137.4
3.85	57.5	3.24	140.4
1.9	66.9	3.56	138.2
2	80.8	3.84	140.2
2.02	46.2	3.61	142.4
2.5	263.7	4.76	120.1
2.48	77.5	3.75	140.9
1.76	64.1	4.1	138.2
2.25	50.1	3.8	139.5
2.45	81.2	4.1	142.8
1.94	70.4	3.54	140.4
3.94	55.2	4.23	131.7
2.37	79.1	4.84	135.4
1.5	79.7	3.97	136.5
1.7	57		130
1.8	58.6	3.4	137.3

3.36	52.9	3.69	137.3
1.63	41.1		144.4
1.64	76		138.8
1.98	79.8	2.87	138.7
1.9	79	3.88	138
1.4	56	4.45	129
1.7	49		143.2
1.9	70	2.27	127
1.4	91	4.2	136
1.5	48	3.8	137
3.58	60	3.33	143
2.05	80.4	3.95	133.1
2.1	84	4.62	141.9
2.37	57.8	3.78	139.3
1.85	62.7	4.05	136.5
1.87	64	3.68	139.5
3.28	77.4	3.22	138.6
2.38	61.4	2.81	143.2
4.14	70.4	4.28	128
1.6	85.5	3.55	134.8
1.78	46.7	3.96	138.8
1.9	74	4.09	146
2.14	91	3.95	135.4
2.09	69.7	3.49	138.1
2.17	58.6	3.64	143.8
1.78	54	4.18	135.8
2.22	73.6	4.02	139.2
1.55	74.7	4.17	137.9
2.78	56.2	4.09	128.4
2.86	60.8	2.63	130
2.29	86.2	4.07	137.1
2.98	55.1	3.72	139.1
2.08	80.7	3.98	143.6
2.21	120.8	3.76	143
1.99	60	3.88	143.4
2.34	33.3	3.46	137.8
2.18	75	3.1	138.8
1.91	70.1	3.49	137.2
2.95	41	2.86	137.9
2.04	74.9	4.13	140.3
1.79	56.2	3.65	137.3
1.84	52	4.37	142

1.78	65.1	4.2	139.7
1.66	73.5	4.29	139.5
2.04	48.3	3.46	139.1
1.65	63.2	3.79	135.6
3.05	59.7	2.96	125.9
1.4	67.1	3.74	138.4
1.79	68.9	4.56	139.7
2.06	59	4.77	135.2
2.56	69.6	3.53	137.2
2.99	74	3.68	142.5
1.8	72	3.37	145.9
2.44	64	3.39	139.6
3.47	72.4	4.22	137
2.96	94.9	3.12	135.7
2.6	52.7	4.24	136.3
1.93	79.2	4.62	138.6
1.63	48	3.22	139.1
1.49	96	3.65	134.3
4.41	290	4.07	133.8
2.28	159	3.95	135.7
1.71	68.8	3.76	140
1.63	64	4.19	143.4
1.61	52	3.82	138.9
3.97	106.5	3.38	129.2
1.83	83.7	4.55	138.1
2.14	47.4	4.7	135.1
1.92	71.7	3.39	137.2
1.91	68	3.93	133.5
1.87	83	3.74	141.3
1.96	173	3.07	136.1
1.92	83	3.79	141.3
2.93	59	3.63	139.4
1.75	54.9	3.09	137.1
2.46	82.8	3.97	137.5
1.74	58	3.4	133.7
2.4	57.4	4.06	135.4
1.78	65	4.21	141.9
4.59	100.3	3.32	138.2
4.3	236.7	3.89	140.1
2.4	61.4	3.67	139.4
2.41	58.9	4.3	140.2
2.3	89	4.74	116.3

2.12	149.7	3.45	128.4
3.33	54	3.45	134.2
1.83	54.9	4.07	136.6
1.97	59.8	3.58	139.4
1.85	63	4.35	137.3
2.58	51	4.65	118.6
1.83	65.3	3.87	133.9
2.27	62.7	3.16	134.3
2.03	79.3	4.4	140.1
3.21	45.6	3.5	140.2
1.91	53.4	4.14	144.6
1.23	65.4	4.01	138.7
3	91	4.93	139.7
2.31	130.2	4.36	126.9
1.86	69.3	4.23	142
4.38	63.4	4.29	138.8
1.15	48	2.79	146.1
1.95	81.3	3.66	138.9
1.7	40.9	4.09	136.8
1.56	79.8	4.75	130.2
1.53	62.3	3.21	134.6
1.51	48.7	4.28	134.6
1.57	61	4.55	141.7
2	38	3.91	137.3
1.66	49.2	2.81	106.6
1.61	63	3.54	140.5
1.8	76.4	3.56	144.1
1.85	69.5	4.56	135.2
1.56	60.6	5.48	140.4
1.64	64.5	3.78	137.3
1.56	84.6	3.57	137.8
1.53	68.8	3.81	136.8
2.47	77	4.1	138.9
